# Applications of CMOS Devices for the Diagnosis and Control of Infectious Diseases

**DOI:** 10.3390/mi11111003

**Published:** 2020-11-13

**Authors:** Saghi Forouhi, Ebrahim Ghafar-Zadeh

**Affiliations:** Biologically Inspired Sensors and Actuators (BioSA), Department of Electrical Engineering and Computer Science (EECS), Lassonde School of Engineering, York University, Toronto, ON M3J 1P3, Canada; egz@cse.yorku.ca

**Keywords:** infectious disease, CMOS technology, point-of-care, lab-on-chip

## Abstract

Emerging infectious diseases such as coronavirus disease of 2019 (COVID-19), Ebola, influenza A, severe acute respiratory syndrome (SARS) and Middle East respiratory syndrome (MERS) in recent years have threatened the health and security of the global community as one of the greatest factors of mortality in the world. Accurate and immediate diagnosis of infectious agents and symptoms is a key to control the outbreak of these diseases. Rapid advances in complementary metal-oxide-semiconductor (CMOS) technology offers great advantages like high accuracy, high throughput and rapid measurements in biomedical research and disease diagnosis. These features as well as low cost, low power and scalability of CMOS technology can pave the way for the development of powerful devices such as point-of-care (PoC) systems, lab-on-chip (LoC) platforms and symptom screening devices for accurate and timely diagnosis of infectious diseases. This paper is an overview of different CMOS-based devices such as optical, electrochemical, magnetic and mechanical sensors developed by researchers to mitigate the problems associated with these diseases.

## 1. Introduction

The accurate and timely diagnosis of infectious diseases such as human immunodeficiency virus (HIV), Ebola, MERS, SARS, COVID-19, tuberculosis and influenza A is indispensable for controlling public health. Early and accurate diagnosis and control of these diseases are necessary to reduce mortality, morbidity and preventing their development into epidemics [1].

Despite the existence of numerous diagnostic systems, current technologies have failed to meet some needs like PoC clinical settings, high throughput screening, rapid tests and high-resolution detection of different pathogens (such as bacteria, viruses and parasites). Additionally, most of the traditional well-established laboratory techniques require large volumes of input samples and biochemical reagents, high accurate biosensors and trained personnel performing assays. Furthermore, sample collection from patients, transferring them to centralized laboratories to be analyzed and sending the test results back to clinicians are sometimes so expensive and time-consuming procedures that patients might lose their opportunity for treatment [1].

**Figure 1:**
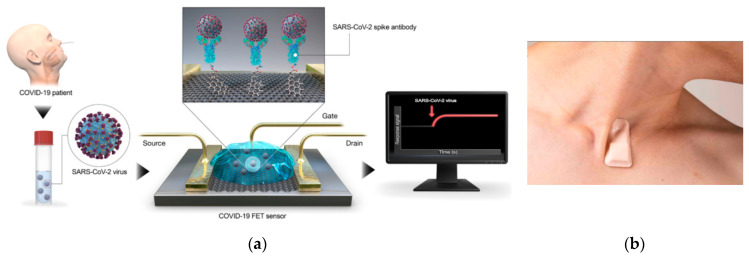
Some of the reported devices for the detection of SARS-CoV-2 using different technologies: (**a**) The graphene-FET based biosensor reported in [2] for the detection of SARS-CoV-2 spike protein and SARS-CoV-2, (**b**) Wireless, soft, skin-interfaced sensor platform for mounting on the suprasternal notch and continuous tracking of key symptoms of the disease [16].

The outbreak of COVID-19 has highlighted the need for rapid and high precision devices to control infectious diseases and as seen in Table 1, various groups made effort to meet these requirements using different technologies. Some groups [2,3] took advantage of graphene field-effect transistors (Gr-FETs) such as the device shown in Figure 1a where the gates of the transistors are made up of graphene sheets. Cardean Bio Inc. has also announced its interest to extend its CRISPR chip for COVID-19 testings and target two genome sequences of SARS-CoV-2 (*E* gene and *RdRP* gene) simultaneously, utilizing a multiplexed microfluidic chip. Its CRISPR-chip is a nucleic acid amplification-free biosensing system which is developed with its proprietory graphene-based biology-gated transistor or Cardean transistor [4,5,6,7]. A printed circuit board (PCB)-based electrochemical device is also reported by Mahari et al. [8] employing disposable screen-printed carbon electrodes. A nucleic acid amplification free optical biosensor is presented by Qui et al. [9,10] combining localized surface plasmon resonance (LSPR) and plasmonic photothermal (PPT) effect. Besides, MEMEMICS Biotechnologies Inc. declared the potential of its Bluetooth-connected PoC device to detect severe acute respiratory syndrome coronavirus 2 (SARS-CoV-2) and associated antibodies from nasal swabs or whole blood [11,12]. Roswell Biotechnologies Inc. and Imec have also announced their partnership to develop a sensor for DNA sequencing which is compatible with modern CMOS chips [13]. In another effort, Cepheid which is well-known in the MEMS community developed a real-time reverse-transcription polymerase-chain-reaction (RT-PCR) test cartridge named Xpert Xpress SARS-CoV-2 [14,15]. Jeong et al. [16,17] claimed to make the first wearable device (illustrated in Figure 1b) which can continuously track key symptoms of the disease such as cough frequency, sound or intensity as well as respiratory rate. Additionally, some smartphone-based devices [18,19,20,21] have been designed for controlling this disease.

Among the new technologies, CMOS technology has opened a new avenue to integrate electronic functions of laboratory equipment as well as arrays of large numbers of biosensors and actuators along with their readout, control and other associated circuitry in a portable, low-cost and disposable device. CMOS technology is a technology for constructing integrated circuit (IC) chips which takes advantage of both n-type and p-type metal-oxide-semiconductor field-effect transistors (MOSFETs). This cutting-edge technology has attracted huge investments for efficient mass production of different platforms including microelectronic devices whose costs can be affordable for the end-users. As seen in Table 2, CMOS technology has many advantages that can overshadow its demerits. High levels of integration with high signal-to-noise ratios (SNRs) due to the reduced external noises and parasites, excellent temperature stability and extremely large fan-out capability are some of the benefits of this technology. Although it is slower than bipolar technology, its power consumption is lower and has a good speed to power ratio [22,23]. 

Furthermore, CMOS technology offers the striking features of high accuracy, short time workflow, multiplexing and parallel detection of a series of parameters as well as high throughput measurements of infectious agents. Also, a CMOS sensor with a micro- or nano-electrode array can detect pathogens with a low limit of detection (LoD) and paves the way for accurate diagnosis of diseases. For instance, an LoD of 10–100 colony-forming unit (CFU) is sometimes required for the diagnosis of infectious agents like dangerous bacteria and CMOS technology enables to implement microelectrodes with almost the same size as the agent, (for example 1µm for bacteria) which helps to detect or count it in an appropriate range of LoD [24].

A growing body of literature [25,26,27,28] has studied the applications of CMOS technology to fight against infectious diseases. CMOS-based PoC devices are among the most useful platforms which can make testing procedures more patient-friendly, less expensive and even more efficient. Specifically, the PoC test platforms utilizing LoC technologies can measure various biomarkers automatically and even simultaneously in less time than laboratory-based tests [29]. Additionally, using CMOS technology along with microfluidics and MEMS allows for sample preparation, actuation, biosensing, data processing and control on a portable and fully automated platform. Furthermore, the required electronic instruments for PoC tests can be adapted to mobile or resource-limited settings and the health-related data obtained using such devices can be collected and sent to health centers through the internet. 

After the outbreak of some infectious diseases like SARS and COVID-19, border-control devices like infrared thermography (IRT) systems have also attracted the attention of some researchers to rapidly mass-screen febrile infected individuals without contact [30]. CMOS technology can help to increase the accuracy of these systems.

Several molecular- and cellular-based assays have been developed for the detection of infectious agents. Popular techniques for detecting pathogens are culture and colony counting, polymerase chain reaction (PCR) and immunological methods like enzyme-linked immunosorbent assay (ELISA) [31,32]. Culturing is the oldest and most reliable technique which is time-consuming and requires trained personnel to complete the test procedures. Additionally, DNA detection is an important molecular method that has been widely used for the diagnosis of infectious diseases and PCR is a popular technique in this field which is based on the extraction of a very small sample of DNA strands and selective amplification of them using specific primers. This technique requires thermal cycling and annealing steps for pre-enrichment and manipulation of DNA [33]. The requirement of a trained person, non-portability, expensive device and difficulties in the quantification of products are some of the limitations of PCR technique which have led to the development of quantitative PCR (qPCR) and real-time PCR to both amplify and detect a DNA, simultaneously. These techniques have the advantages of minimal amount quantification, real-time amplification and rapid detection [27]. 

Immunological techniques are other biochemical tests useful for the measurement of the presence or concentration of a pathogen in a solution through the use of the selectivity of antibody-antigen interaction. The combination of these techniques and other sensory systems can provide a complete self-contained detection package [33]. For example, ELISA systems, which are widely used for bio-clinical applications, take advantage of the sensitivity of enzymatic detection and selectivity of antibodies [31]. A photospectrometric instrument in these systems enables to analyze the colorimetric change caused by the concentration of target bio-particles affected by the immune reaction. Despite its high sensitivity, specificity, multiplexed and high throughput properties, this technique is a step-by-step laborious procedure requiring expert knowledge and a significant amount of time for sample preparation and analysis. Alternatively, immunochromatography, as a combination of chromatography and immunoassay, is a rapid, one-step assay [34]. Some of these approaches can be miniaturized and even improved by using advanced CMOS-based sensors.

This paper is an overview of various CMOS-based devices reported for the diagnosis and control of various infectious diseases (arising from different air-, vector-, blood-, and food-borne infectious agents). Various types of biosensors have been developed for biological and chemical sensing which can be useful for the diagnosis of infectious agents. These biosensors might be based on optical [35,36], electrochemical [37,38], magnetic [39] or mechanical [40] sensing modalities which can be developed by CMOS technology. 

The rest of the paper is organized as follows. The second section is dedicated to the applications of CMOS-based optical devices for the diagnosis of infectious diseases. In the third section, different types of CMOS-based electrochemical biosensors reported for these applications are reviewed. The other biosensors like magnetic and mechanical biosensors implemented by using CMOS technology are discussed in the fourth section followed by a conclusion in the final section.

## 2. Optical Techniques

Optical sensing is the most popular technique for biosensing applications due to their reusability, rapid measurement and high sensitivity. As seen in Table 3, various CMOS-based optical devices have been reported for the diagnosis of different infectious agents. 

Development of CMOS image sensors (CISs) has led some authors like Wang et al. [41], Pérez et al. [42], Bishara et al. [43], Moon et al. [44], sun et al. [30] and many others to investigate the application of CMOS-based imaging techniques for detecting the agents or even the symptoms of infectious diseases. In comparison to other image sensors like charge-coupled devices (CCD), CISs can be integrated with other processing circuits, analog-to-digital converters (ADCs) and control systems. Although the quality of the image captured by CISs falls short of that by CCD sensors, some successful experimental results prove that CISs can provide high sensitivity, high specificity, low power dissipation, operational simplicity, short time to results and cost-effectiveness [45]. Additionally, CMOS technology allows substituting expensive laser sources with more economic light-emitting diodes (LEDs). 

It is also noteworthy that smartphones are powerful platforms to develop portable, low-cost, and readily accessible alternatives to conduct a variety of detection tests [35,46,47,48,49], especially due to their high-quality and multifunctional camera which can detect chemiluminometric, colorimetric or fluorometric signals produced from biosensors.

However, most optical techniques require labeling processes that are cost- and time-consuming and in some cases might affect the interaction between target molecules and the probe [50]. Also, most optical biosensors need costly and bulky instruments such as optical scanner systems consisting of light sources, lenses and cameras hindering the miniaturization of the systems [40].

The last few years have witnessed a huge growth in the development of various compact on-chip imaging approaches such as holographic microscopy, contact shadow imaging, fluorescent on-chip microscopy which can replace conventional lens-based microscopes and be more appropriate for LoC applications [51]. Some of the reported optical techniques have removed the need for labeling in in-vitro diagnosis.

### 2.1. Luminescence Imaging for In-Vitro Diagnosis

Fluorescence and chemiluminescence are among the most reported luminescence techniques for pathogen detection. The former is triggered by light and the latter is triggered by a chemical reaction. 

In fluorescence, after stimulation of a substance by introducing an excitation light to it, the electromagnetic radiation is absorbed and subsequently, a light of a longer wavelength is emitted for a very short time [66]. Fluorescence-based biosensors work based on monitoring the frequency changes of this radiation [66]. There are three types of fluorescence sensing: (1) direct sensing in which specific target biospecies could be detected directly before and after a change or reaction without a labeling tag which requires a high-speed image sensor; (2) indirect biosensing in which an added dye (like green fluorescent protein) helps to transduce the presence of the target; (3) fluorescence energy transfer (FRET) in which two paired fluorophores with overlapped emission and excitation wavelengths are placed within about few Angstroms from each other and the excitation of one of them stimulates the fluorescence of the pairing one and a unique fluorescence signal is generated [66,67]. 

**Figure 2:**
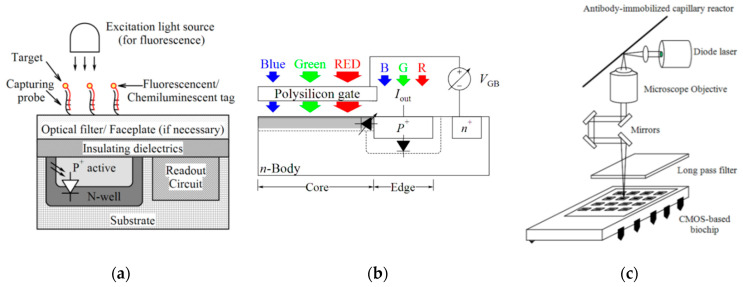
(**a**) Fluorescence/chemiluminescence imaging, (**b**) Cross-sectional view of a filterless CMOS color photogate sensor, (**c**) Schematic diagram of a compact CMOS biochip immunosensor for the detection of a single bacteria.

As shown in Figure 2a, a transducer such as a photo-detector transduces the emitted signal in fluorescence detection to an electrical signal like voltage or current. The photo-detector can be implemented by an embedded PN-junction in CMOS technology. For the integration of the fluorescence module into a CMOS chip, the metal layers above the photo-detector should not block the optical signal. Also, if the intensity of the excitation signal is stronger than the fluorescent signal, it can lead to the saturation of the photo-detector and malfunction of the system [67]. Jang et al. [68] used a thin-film long-pass optical filter atop the CMOS chip to filter the excitation signal and a fiber-optical faceplate to direct the signal. Ta-Chien et al. [69] proposed a time-resolved fluorescence detection by the adoption of a time-gated arrangement without any external filters. In another effort, Hong et al. [70] reported a fully integrated CMOS fluorescence biosensor with an on-chip nanophotonic filter.

Ho et al. [53] developed a spectrally-multiplexed fluorescence contact imaging microsystem for DNA analysis. In this system, a CMOS color photogate sensor (see Figure 2b) utilizing the polysilicon gate as an optical filter is integrated into the microsystem and detects and differentiates among multiple emission bands (green and red light) of fluorescent biomarkers without a bank of emission filters. The capability of multi-color imaging led to the simultaneous detection of two DNA targets with the LoD of 240 nM and 210 nM for the marker gene sequences of spinal muscular atrophy disease and *Escherichia coli* (*E. coli*), respectively (see Table 3). 

In another effort, Song et al. [52] reported a field-usable biochip system as shown in Figure 2c which works based on a miniature CMOS sensor microarray by combining ELISA and laser-induced fluorescence (LIF) techniques to detect a single intact *Bacillus globigii* (*B. globigii*) spore. An antibody-immobilized capillary reactor was used to detect bacteria. Enzymatic amplification following immunocomplex formation removed the need for a bulky optical system to achieve high sensitivity levels. As seen in Figure 2c, a diode laser beam that is focused onto the detection window of the antibody-immobilized capillary reactor by a lens, excites the enzymatic product. Multiplex antibody-immobilized capillary reactors increased the high throughput for *B. globigii* spores detection and a side-entry laser beam irradiation allows for the simultaneous excitation of four multiplex antibody-immobilized capillary reactors. The fluorescence emission from the reactor was collected at right angles by a microscope objective lens and focused onto a photodiode of the CMOS microchip. Optical mirrors helped to compact the collected fluorescence emission path and the bandpass filter removed the diode laser scattering.

In another work, Zeinhom et al. [35] proposed a smartphone-based fluorescence imager with a sandwich immunosensor for *E. coli* O157:H7 detection which could provide low-background noise image and good sensitivity. The imager utilized a long-pass thin-film interference filter, a compact laser-diode-based photo source, and high-quality insert lenses. The external lens collected the fluorescence emission from the sample and the CMOS sensor chip and the built-in lens on the cell phone recorded the magnified fluorescent images. Like other smartphone-based devices, this device also needs afterward image processing. The whole process could be completed within two hours and the experimental results demonstrated that this device could provide an LoD down to 1 CFU/mL (Table 3). In another effort, Natesan et al. [48] used a smartphone fluorescent reader and a flow cell assay cartridge for the telemonitoring of infections caused by Ebola and Marburg filoviruses. The reader was utilized to detect and analyze antibody binding to twelve essential antigens from the filoviruses arranged in a microarray on a microfluidic chip. An opto-electro-mechanical hardware attachment in the smartphone reader helped to handle the operation, collect test results and communicate with cloud service.

Lateral flow immunochromatographic assay (LFIA) is a simple technique useful for qualitative and semi-quantitative detection of various biomarkers such as antigens, antibodies and even the products of nucleic-acid-amplification tests. LFIA requires an electronic reader for the detection of low concentrations by reading the intensity of the color test line. The camera of a mobile phone is the most common electronic reader used for LFIA to take a photograph of the strip and the quantification of the analyte in the sample. Different camera specifications as well as standardization and regulatory issues cause some limitations in the use of mobile phone-based readers. Additionally, the transmission of the images captured by image sensors through a data cable results in a poor quality of the images due to being exposed to air. 

**Figure 3:**
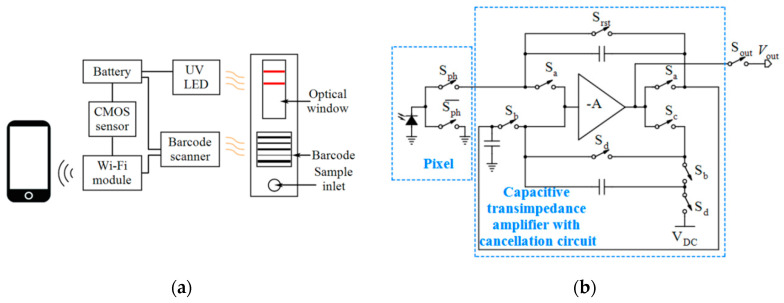
(**a**) Hardware structure of a strip reader, (**b**) The pixel and processing architecture of an LFIA reader.

In an effort, Zheng et al. [54] used a CMOS transistor as the image sensor and developed a portable test strip reader (see Figure 3a) for quantum dot (Qdot)-labeled urea-enzyme antibody-based rapid immunochromatographic test strips to detect *Helicobacter Pylori* (*H. pylori*). The reader is integrated with a Wi-Fi module and can be run on tablet PCs without the need for an AC power supply. The 3D printed cartridge of the LFIA was used for this reader. A barcode scanner loaded the barcode printed on the strip containing the patient information and the acquired images were processed by a Kmeans clustering algorithm. Another approach is using a single photodiode at the position of the test line. However, this approach is susceptible to displacement errors. Pilavaki et al. [56] reported a CIS for LFIA readers containing 4 × 64 pixels in contrast to a mobile phone image sensor with million pixels. This reader takes advantage of a single low power processing capacitive transimpedance amplifier with noise cancellation (Figure 3b). Low power consumption (21 µW), low total output referred noise (1.9 mV_rms_) and high SNR are some main features of this reader. Additionally, this system does not require any moving parts and optical accessories. Experiments showed that this LFIA system is able of detecting influenza A nucleoprotein with concentrations from 0.5 ng/mL to 200 ng/mL (see Table 3).

**Figure 4:**
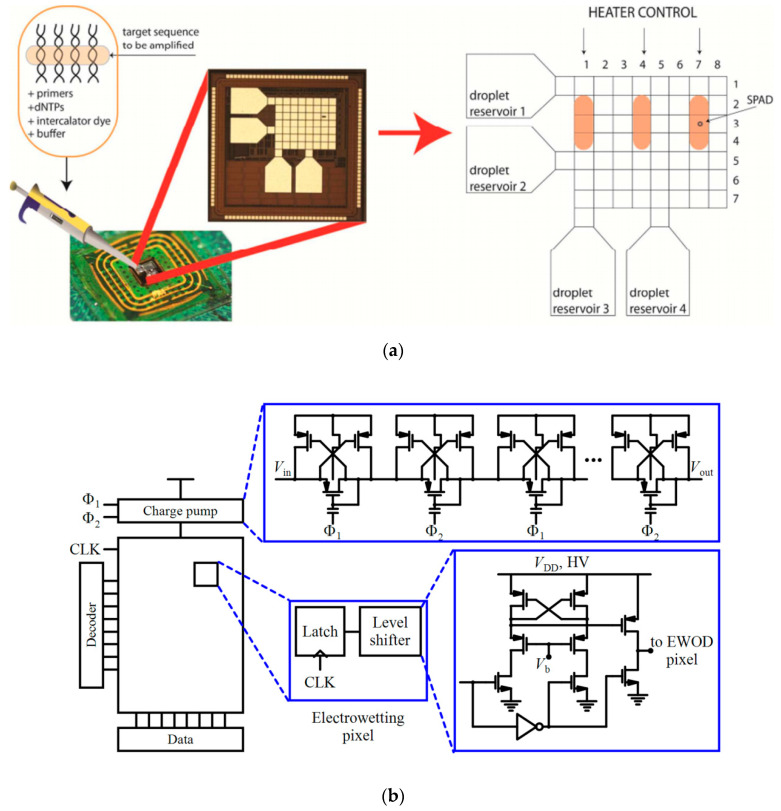
(**a**) The CMOS qPCR LoC containing a 7 × 8 array of electrodes for EWoD droplet transport [28], (**b**) The electrowetting control circuitry and the custom level shifter interfaces used in the system shown in (**a**).

In addition to some CMOS-compatible PCR chips reported in the literature [71,72], some scientists have focused on doing qPCR approach by using CMOS-based devices. For example, Norian et al. [28] used microfluidics and 0.35 µm high-voltage CMOS technology to perform all the functions required for qPCR including fluorescence detection, heating, temperature control and steering the fluid sample. Electrowetting-on-dielectric (EWoD)-based droplet transport, micron-scale polysilicon heaters and resistive aluminum (Al) temperature sensors were used instead of manual pipetting, Al heating block and a thermocouple, respectively. Additionally, integrated Geiger-mode single-photon avalanche diodes (SPADs) were utilized for the fluorescent detection of the PCR reaction. To perform qPCR, droplets of primers, DNA target and the PCR reagents (DNA polymerase, deoxynucleoside triphosphates (dNTPs), and intercalator dye) were delivered to individual reservoirs (see Figure 4a) and electrostatically drawn from each of them and mixed. The thermal cycling of the entire surface of the chip was based on a prescribed heating profile. For example, in the heating profile shown in Figure 4a, fluorescence reading after qPCR progression is taken using an integrated SPAD at Pixel (3,7). Figure 4b also shows the EWoD control circuitry. In this system, the temperature can be regulated to an accuracy of 0.45 °C and droplet generation and transport can be down to volumes less than 1.2 nanoliters. Dynamic range and sensitivity allow a single copy per droplet for the quantification of target DNA. *Staphylococcus aureus* (*S. aureus*) was used to demonstrate the performance of this device.

Loop-mediated isothermal amplification (LAMP) as an alternative to the conventional PCR is suitable for the development of inexpensive, rapid, low power and portable systems with greater sensitivity and specificity for PoC diagnosis. Among various isothermal amplification methods, LAMP is a sequence-specific amplification technique that eliminates the need for the thermal cycler by utilizing a single displacing DNA polymerase enzyme. Shin et al. [46] combined different elements of the mobile phone hardware as well as droplet magnetofluidics for complete process integration of a sample-to-answer nucleic acid amplification testing assay from sample lysate to detection. They developed a magnetofluidics-assisted LAMP assay for nucleic acid target amplification and used magnetic particles as a mobile substrate to capture and transport nucleic acid. An embedded CMOS camera sensor is employed in this platform for optical signal acquisition, in which an LED illuminates the reaction chamber and the generated fluorescent signal by the reaction is relayed by a mirror and filtered and then magnified by a combination of an embedded camera lens on the phone and an external imaging lens before reaching the app-based user interface. Additionally, Bluetooth connectivity allows for wireless communication with the instrument’s on-board microcontroller to automate and coordinate magnetofluidic actuation and thermal incubation. 

In another effort, Priye et al. [47] used a smartphone CMOS sensor to quantify fluorescence-based nucleic acid amplification. Their image processing pipeline used chromaticity-luminance color space to measure the luminance of fluorescent signals arising from nucleic acid amplification targets and separated the color aspect of the image and the luminance values. This enabled manual control over the intensity of the collected signal. This chromaticity-luminance formulation was verified by simultaneous detection of chikungunya and Zika viral RNA via endpoint reverse transcription LAMP (RT-LAMP) as well as real-time LAMP detection of *Neisseria gonorrhoeae* (*N. gonorrhoeae*) and demonstrated more reliability and accuracy than a conventional post-processed RGB (Red, green, and blue) analysis.

In chemiluminescence, the chemiluminescent tags avert the external light source to excite the chemical reaction and prevent the saturation of the photodiode. So, a photodetector can detect the emitted chemiluminescent signals without an optical filter and the distance from the tag to the surface of the photodetector can be shortened enabling supercritical angle luminescence and efficient signal detection [67,73]. Jeon et al. [25] integrated a lens-free CIS with a cellulose membrane-based immunosensing platform, ELISA-on-a-chip (EoC), for the detection of *Salmonella typhimurium* (*S. typhimurium*). Two image sensors were mounted on a PCB which could be connected to a signal detection system. In this platform, the chemiluminescent signals were produced from the EoC and after being transferred into the image sensors, they were converted to electric signals on the detector. A traceable amount of the bacterium (4.22 × 10^3^ CFU/mL) can be detected by this sensor. Furthermore, an immuno-magnetic separation device was introduced for the enrichment of the bacterium before the analysis which resulted in an additional 67-fold enhancement in the LoD (see Table 3). 

**Figure 5:**
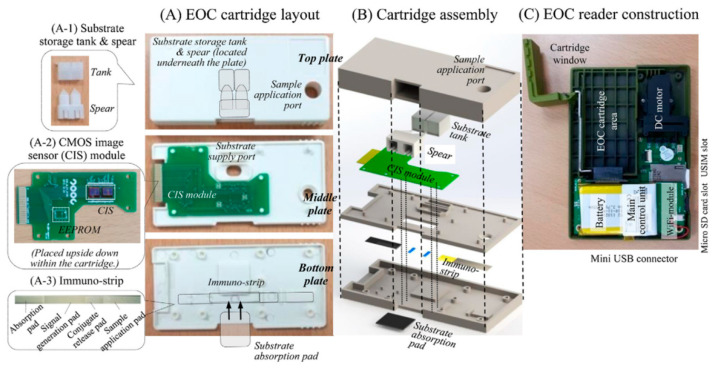
Cartridge layout of an EOC and its assembly, and the EOC reader construction proposed in [49].

The same group [49] used a CIS to fabricate a pocket-sized immunosensor for the detection of the *Vibrio* species, in a real sample. A smartphone was used to control and monitor the analysis and upload the results as data to the internet server. This study is an exemplification of food-born pathogen monitoring via internet-of-things in the field of healthcare. Figure 5 shows the cartridge layout of this EoC consisting of three plastic plates and its assembly, as well as the EoC reader construction. The top plate includes a port to apply the sample to the substrate storage tank located underneath and the immuno-strip on the bottom plate. The tank punches the membrane with spears to supply the substrate solution at the time of signal generation. Two delivery passages on the middle plate steer the sample and the enzyme-substrate to the bottom plate. Additionally, the middle plate includes the sensing module consisting of two CISs and electric circuits on PCB. The immuno-strip and the substrate absorption pad are placed on the bottom plate. The EoC reader controls analysis, processes the signal, and transmits the result. Although the sample incubation before analysis is time-consuming which may limit the application, running mobile safety laboratory can resolve this limitation and be effective in controlling the spread of infectious diseases and management of the data generated by various wearable biosensors for healthcare via internet-of-things. In another effort, a simple procedure is reported by Baader et al. [55] to create polysaccharide microarrays for analyzing antibodies with a CMOS-based electric signal readout process and using fluorescence or chemiluminescence based detection. This chip includes an array of silicon photodiodes. Unmodified pneumococcal polysaccharides from *S. pneumonia* are directly printed onto photodiode surfaces which is helpful for the measurement of anti-polysaccharide IgG antibodies in human blood serum.

### 2.2. Label-Free Imaging for In-Vitro Diagnosis

**Figure 6:**
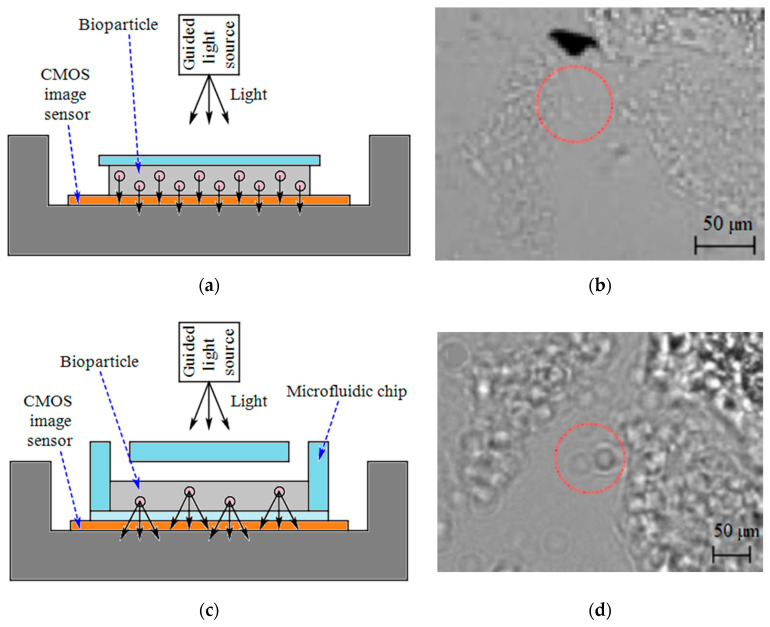
(**a**) Direct contact imaging, (**b**) Direct contact images of *E. coli* imaged with a 5.2 µm pixel CIS [57], (**c**) Shadow imaging method, (**d**) Diffracted shadow images of *E. coli* imaged with a 2.2 µm pixel CIS [57].

Larger biological objects, such as bacteria and cells can be detected directly without a labeling tag by using high-speed CISs. To remove expensive and bulky optics like mirrors and lenses, some lensless techniques like direct contact imaging, shadow imaging and lens-free holographic microscopy were developed. Contact imaging (see Figure 6a) helps to image the object close to the focal plane with the resolution in the order of the pixel size without the need for expensive and bulky optics such as a system of lenses and mirrors in the conventional fluorescent microscopes.

Shadows of small objects are also used for holographic microscopy and on-chip shadow imaging [74]. In shadow imaging, any optically semi-transparent micro-object such as cells is directly placed on top of an optoelectronic image sensor and illuminated by an incoherently or partially coherent light source. The generated diffraction patterns give useful information about internal complexity, shape, refractive index or size of the targets. This technique provides the advantage of high throughput monitoring since it does not optically magnify the original optical plane where the target micro-objects are placed and can fully monitor the whole active imaging area [58]. Figure 6a,c compare the direct imaging method and shadow imaging one by simple schematics. Figure 6b,d demonstrate the images of *E. coli*, as a model organism, which are captured by a 5.2 µm-pixel and a 2.2 µm-pixel CIS, respectively [57]. The magnification caused by the diffraction effect of the shadow spreading is obvious in Figure 6b,d. In comparison to shadow imaging in which the diffraction between the detector planes and the object is ignored or unprocessed, lens-free holographic devices used digital refocusing of object waves to image an object plane regardless of optical diffraction. The required lenses in traditional microscopy are actually replaced by a computer algorithm in holographic microscopy to reconstruct the original image from the recorded hologram [51]. 

Moon et al. [44] integrated microfluidics with a shadow imaging technique to target CD4^+^ T-lymphocyte counts for HIV PoC testing from whole blood (see Table 3). This platform can capture rare cells selectively by the immobilized anti-CD4 antibody on one side of the microfluidic chip, detect the captured cells rapidly by the imaging platform, and count cells automatically and enumerate CD4^+^ T-lymphocytes. Also, the same group [57] reported a lens-free imaging platform like Figure 6c to enumerate cell/*E. coli* to diagnose sepsis at resource-limited conditions. This platform can directly image the bottom surface of a microfluidic channel and shadow dots of captured cells are counted by automatic cell counting software. The smaller sizes of bacteria (diameter of 5 µm × length of 2 µm for *E. coli*) make seeing their shadows more challenging than cells. The experiments showed the functionality of the platform to capture the target cells from 5 µl of blood by using specific antibodies and imaging *E. coli* by employing a 2.2 µm-pixel CMOS based imaging sensor (see Table 3).

A lens-free CIS is proposed by Lee et al. [58] for the detection of pathogenic bacteria, such as *Listeria monocytogenes* (*L. monocytogenes*) and *S. typhimurium*, which works based on shadow imaging and detects the colorimetric light signals in the ELISA process. It is a compact platform whose major components are an LED and a CIS. To achieve uniform illumination, a pinhole mask formed on a transparent plastic film is glued on top of the LED window. A commercial 96-well plate is located between the CIS and LED. A custom-made aperture cover with a 2 mm diameter hole array is located on top of the 96-well plate to minimize noise signal. Employing a multi-million-pixel photon collector led to a decrease in the noise signal from noise sources like light scattering through the transparent well plate walls, the dark current of the photon detector and unwanted particles in the sample solution.

Devadhasan et al. [60] reported an immunodiagnostic system for detecting HIV which works based on HIV antigen-antibody reaction adsorbed on an indium nanoparticles (InNPs) substrate and photon counting using a CIS. The InNP substrate as a semi-transparent material can pass the photons consistently. The CIS captured the refracted photons by the InNP substrate while passing through it. This technique could overcome the non-specific interaction in label-free diagnostic systems. As seen in Table 3, their sensor was capable of detecting HIV antigens less than 10 fg/mL. They used a similar approach in a CIS-based immunodetection system for HBV antigen-antibody reaction on InNP substrate [61]. 

In another effort, the same group [27] presented a disposable CIS integrated with polypropylene wells to carry out LAMP assay for simultaneous and real-time amplification and detection of nucleic acid. The image sensor chip included 376 × 314 multi-pixel photon arrays with dual top headers and was connected to a 10-bit ADC. Two wells were fixed for the two sensor surfaces of the CMOS chip to carry out two parallel experiments which can help to reduce the reaction volume and cost. The image sensor captures the light passing through the sensor surface on top of which the LAMP reaction is carried out. Generally, when the photons are exposed to objects over the CMOS sensor, their number will be changed. The photodiodes in the image sensor capture and convert the photons into electrical energy via the photoelectric effect. The ADC converts the electrical energy to digital. During the LAMP reaction, magnesium pyrophosphate is generated and the LAMP sample color and the photon number are changed. During the amplification process, the photon number is reduced. During the LAMP reaction, the circuitry controller RS-232 provided an appropriate temperature for amplification and standard light intensity for photon detection. The analysis was carried out on the DNA of *Clostridium perfringens* (*C. perfringens*), as the main pathogenic bacteria of enteric disease and gas gangrene (Table 3). As mentioned in Table 3, they achieved an LoD up to 1 fg/µL. In continuation of this work, the same group [41] used *E. coli* O157 (see Table 3) as their target to evaluate LoD and quantitative analysis of their system. Despite its good sensitivity and its potential for PoC assays, the system suffers from some shortcomings. The used red or blue LED as the light source affected the difference in the quantitative results. Additionally, some inconsistency was found with the detection of different analytes. Furthermore, every DNA sample requires a different reaction time [45].

**Figure 7:**
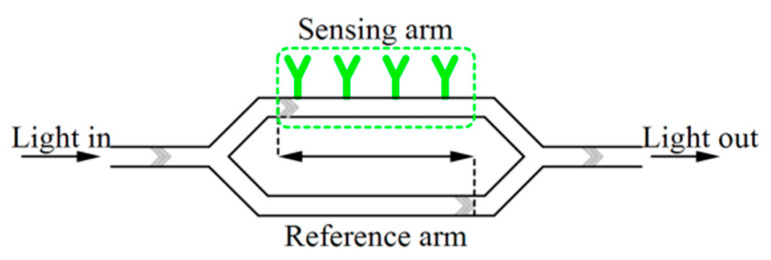
An MZI sensor.

Interferometer-based biosensors are among optical label-free biosensors measuring the change of the refractive index. By propagation of light through an optical waveguide, an evanescent field is created at the core/cladding interface extending into the surrounding area. If the waveguide surface is functionalized by biological recognition elements (REs), the sensing layer will change the refractive index profile [66]. Mach-Zehnder interferometer (MZI) (see Figure 7), Young’s interferometer, Hartman interferometer and Backscattering interferometry are different types of such sensors [75]. For example, Ramirez-Priego et al. [65] used the first type to develop a nanophotonic PoC platform for noninvasive and direct detection of lipoarabinomannan (LAM) in human urine samples for the diagnosis of tuberculosis. This sensor which is based on an MZI transducer is combined with an on-chip spectral filter. In a typical configuration of MZI transducer as depicted in Figure 7, there are two branches one of which is functionalized and called the sensing arm and the other works as the reference arm. A Y-junction divides the guiding light into these two branches and another Y-junction recombines them. Then, the interference of the two branches with each other produces a sinusoidal change related to the refractive index variation of the surrounding medium. In the photonic sensor chip reported in [65], a superluminescent diode (SLED) and a CMOS sensor were used as the light source and the signal readout device, respectively. Also, a graphical user interface and a pumping unit allow for real-time monitoring and controlling of fluid injection through the sensor cartridge. Monoclonal antibodies were functionalized against LAM to achieve suitable selectivity.

In another work, Daaboul et al. [64] used a single-particle interferometric reflectance imaging sensor (SP-IRIS) to automate sizing and counting of thousands of individual virions. Different imaging wavelengths (like Violet/UV and green light illumination) enable to visualize various sized particles including small (40 nm), medium (100 nm), large (400 nm), and filamentous (90 × 1000 nm) virus particles like Zika virus, vesicular stomatitis virus, vaccinia virus, and Ebola virus.

**Figure 8:**
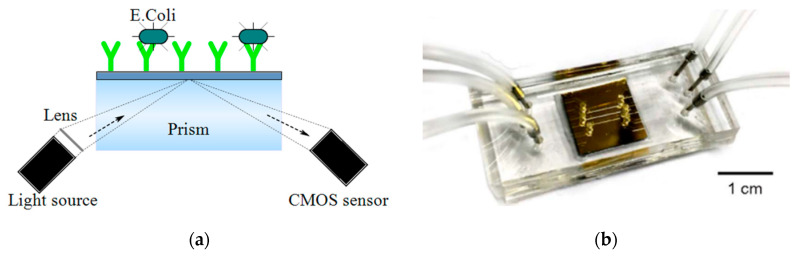
(**a**) Schematic of an SPR platform using a prism, (**b**) The gold plasmonic chip assembled with the PDMS microfluidics reported in [63].

Surface plasmon resonance (SPR) is the resonant oscillation of conduction electrons occurring on the surface of conducting material (like metal) at the interface of two media with dielectric constants of opposite signs which is illuminated by polarized light at a specific angle. This phenomenon generates surface plasmons and reduces the intensity of reflected light at a specific angle [75,76]. The refractive index variation induced by molecular interactions is related to surface density or sample concentration which is useful for label-free detection [75]. If an RE is immobilized on conducting material surfaces to capture specific biospecies, the LoD of the system is influenced by the refractive index of the target, sample volume and transport properties, target immobilization method and practical device parameters [62]. There are four basic techniques to excite the SPR: (1) prism coupling, (2) waveguide coupling, (3) optical fiber coupling and (4) grating coupling [75]. For example, Tokel et al. [62] proposed an SPR platform using a rectangular prism (like Figure 8a) which was integrated with microfluidics for the detection of *E. coli* and *S. aureus*. A CIS was used to capture the reflected light. In another effort, Wood et al. [36] used a CMOS camera to perform a single-molecule fluorescence assay (FLISA) on plasmonic gratings and analyze tuberculosis biomarkers from urine clinical samples of HIV negative patients with tuberculosis. Additionally, Soler et al. [63] reported a nanoplasmonic biosensor integrated with microfluidics, as depicted in Figure 8b, for simultaneous detection of *N. gonorrhoeae* and *Chlamydia trachomatis* (*C. trachomatis*). This biosensor incorporates gold nanohole sensor arrays and works based on extraordinary optical transmission (EoT) which enables label-free and real-time detection and facilitates high-throughput analysis. Immobilization of specific antibodies on the individual arrays allows for selective detection of bacteria. EoT can be performed by using LEDs and CMOS imagers. Direct immunoassay of urine samples demonstrated an LoD of 1500 CFU/mL for *N. gonorrhoeae* and 300 CFU/mL for *C. trachomatis*.

**Figure 9:**
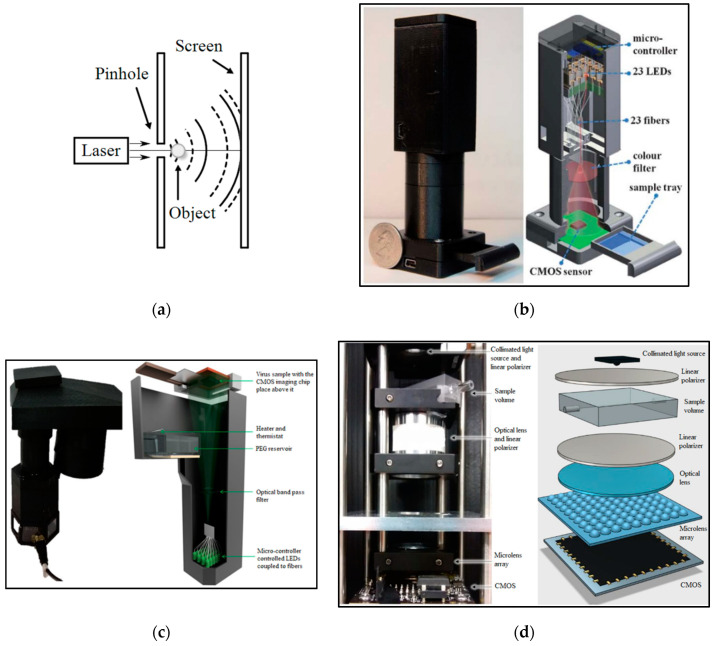
(**a**) Inline holographic microscopy, (**b**) The photograph and schematic of lens-free super-resolution microscope using a fiber-optic array reported in [43], (**c**) The photograph and schematic of the portable lens-free microscope reported in [59], (**d**) The photograph and schematic of the image cytometer based on angular spatial frequency reported in [42].

Numerical reconstruction of digitally recorded holograms has paved the way to study µm-sized 3D phenomena. The contour of the nanometric structures can be determined by using phase information or optical scanning holography. But, all these techniques need complicated optical setups. Digital in-line holographic microscopy is a simpler technique to capture 3D images of objects with µm-resolution and also study moving objects. Figure 9a shows the schematic of a digital in-line holographic microscope in which a spherical wave of a wavelength is provided from a point source (a laser is focused onto a sub-µ sized pinhole) of linear dimensions of the order of a wavelength and illuminates an object. As a result, a highly magnified diffraction pattern is formed on the screen [77]. A high-resolution microscope based on partially coherent digital in-line holography is reported by Bishara et al. [43] including a CMOS sensor, a color filter and 23 LEDs for the detection of malaria parasite infection in red blood cells (see Figure 9b). A microcontroller controls the multiple fiber-optic waveguides, which are butt-coupled to LEDs, to sequentially illuminate the sample. Then, a digital sensor-array captures the holograms whose resolution will be improved by a pixel super-resolution algorithm. The performance of this microscope is verified by imaging human malaria parasites (*Plasmodium falciparum* (*P. falciparum*)) in thin blood smears. 

In another work, Ray et al. [59] reported a computational imaging framework by using both the physical size range and viral immuno-specificity of the viruses to enumerate herpes simplex virus (HSV-1) particles (which are approximately 150–200 nm in size). As shown in Figure 9c, a programmable-array of LEDs coupled to optical fibers illuminates the substrate and a CIS records the hologram created due to the scattered optical wave from the viruses and the non-diffracted wave. Self-assembled polyethylene glycol based nanolenses around the viruses help to boost the optical signal from each virus particle and increase SNR. This platform is able to reconstruct both amplitude and phase information of the objects digitally from the diffraction patterns obtained by the image sensor chip.

Pérez et al. [42] introduced an image cytometer for the detection of pathogenic bacteria such as *E. coli* and *Legionella pneumophila* (*L. pneumophila*) which worked based on the detection of the Fourier transform image of the sample plane. Figure 9d demonstrates the schematic of the system including an RGB fiber-coupled LED light source, a quartz flow cell, two ultrahigh contrast linear polarizers, an optical lens, a micro-lens array and a CMOS color image sensor. In this system, micro-lens arrays filtered the transmitted beam which led to the increased depth of field (DoF) and field of view (FoV). This system can retrieve sample information without complex digital transformations. A sensitivity of 50 cells/mL can be achieved. Pre-concentrating an initial sample volume of 500 mL with an ad-hoc fluidic system can increase the sensitivity to 0.2 cells/mL.

### 2.3. Contactless Monitoring of Diseases’ Symptoms

**Figure 10:**
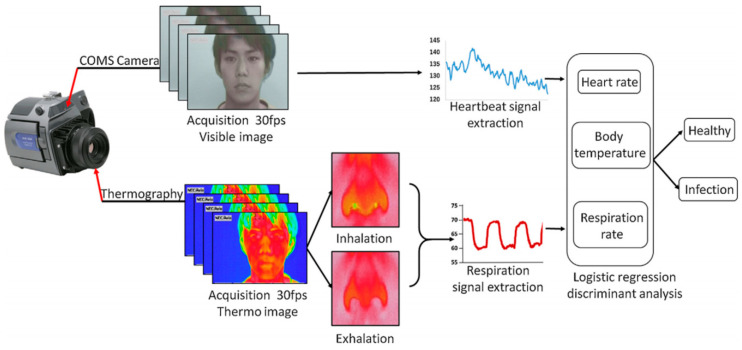
The visible and thermal image processing approach to sense multiple vital signs and the multiple logistic regression function remotely which helps to predict the possibility of infection [30].

Although IRT systems are useful for border control and fever-based screening, several factors like the consumption of antipyretic medications, the environmental temperature and humidity as well as alcohol consumption can influence their measurements. So, they suffer from low sensitivity. The association between infections and inflammation has led to vital-sign-based screening. Matsui et al. [78] used this concept to develop an infection screening radar system to mass-screen individuals remotely. They used a multisensor technique to measure a heart and respiration rates as well as the facial skin temperature. But, their radar system incorporated expensive embedded modules (like a reflective photoplethysmography sensor, a microwave radar, and IRT) and large-scale systems. To minimize the required hardware, they enhanced the functionality of the system which has already been installed at most major international airports. This group [30] developed a CMOS camera-equipped IRT system by the combination of visible and thermal image processing approaches (see Figure 10) and tested it on patients with an influenza-like illness. These systems can remotely sense heart and respiration rates and body temperature. The temperature changes around the nasal area on thermal images can be used to monitor respiration rates. The facial skin temperature is measured simultaneously. The tiny color changes arisen from the circulation of blood in the face can be detected by visible facial image to determine the heart rate. The possibility of infection can be predicted by a multiple logistic regression function incorporated into the system.

## 3. Electrochemical Techniques

Electrochemical sensors have the potential of being integrated by CMOS technology with little or no external (optical) components or post-processing. They are suitable for portable and low-cost platforms as well as real-time and label-free measurements without the need for cumbersome labeling processes [33]. As seen in Table 4, different kinds of electrochemical sensors are reported for the detection of infectious agents including potentiometric [33], impedimetric [79], capacitive [80] and amperometric [24] sensors. In these sensors, when sensing electrodes come to contact with target bioparticles, electrical characteristics of the electrodes like dielectric constant, electrical charge and conductivity are changed that can be converted to readable electrical signals like current, voltage or digital data.

### 3.1. Biological/Chemical Field-Effect Transistors (FETs)

**Figure 11:**
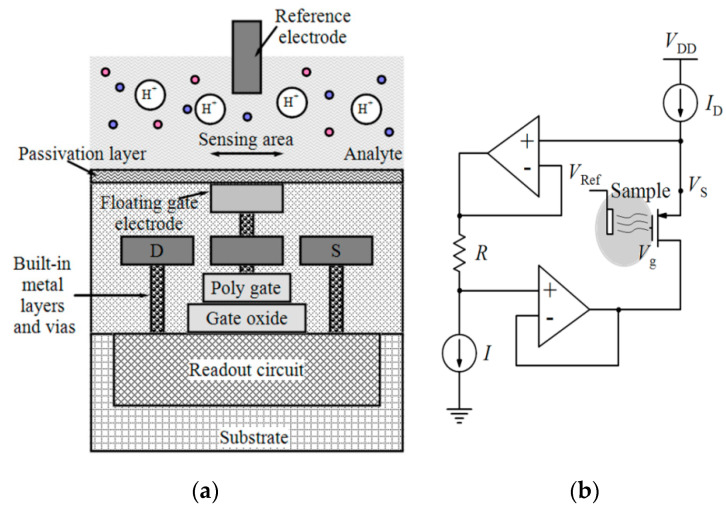
(**a**) ISFET fabricated by standard CMOS process, (**b**) An ISFET readout circuit.

The biological or chemical FETs like ion-selective field-effect transistors (ISFETs) are the most common types of potentiometric sensors which are generally used for the measurement of cell activity (such as potential hydrogen (pH) variations) rather than viability, but they also have been reported for other applications like detection of bacterial activity [93] or bacteria DNA hybridization [82]. The gate electrodes of these kinds of FETs are covered with selective membranes (such as DNA-selective membranes, ion-selective, and enzyme-selective) which can modulate the gate voltage [33]. The traditional ISFETs, introduced by [94], did not have a poly-silicon gate which was not compatible with the standard CMOS process. Bausells et al. [95] adapted the fabrication process of ISFETs to the standard CMOS process (Figure 11a). In these ISFETs, the poly-silicon gate is connected to the top metal and oxynitride passivation layer (as the pH-sensitive material) [83]. It is worth mentioning that ISFET is sensitive to temperature and a power-hungry heater, stable environment and a large reference electrode is required [40].

Rothberg et al. [82] used ISFETs with DNA selective membranes (genetic ISFETs) to detect bacteria DNA hybridization. The chip consists of a large array of ISFETs aligned with 1.2 million wells to confine and detect independent sequencing reactions, simultaneously. In contrast to light-based sequencing technology in which photons are collected by the elements of the array, each sensor of this array directly and independently monitors the hydrogen ions released during the polymerization of DNA strands on microbeads. The performance of this system is demonstrated by sequencing three bacterial genomes (*Vibrio fischeri*, *E. coli* and *Rhodopseudomonas palustris* (*R. palustris*)). 

In ISFET based DNA sequencing, the unknown distribution of DNA-templated microbeads into microwell array can lead to false pH detection at empty microwells affected by the cross-talk from neighboring microbeads. Additionally, a large array of ISFETs may have the pixel-to-pixel threshold voltage mismatch. Huang et al. [83] proposed a 64×64 arrayed dual-mode CMOS ISFET sensor for DNA sequencing including the ISFET and CIS pixels fabricated by CMOS process. The imaging of the microbead distribution is based on the lens-free contact imaging principle which helps to determine the microbead physical locations accurately. So the sensor can correlate the measured pH response with the physical locations containing microbeads and the accuracy of pH detection will be improved. Additionally, they used correlated double sampling (CDS) readout for both modes to reduce pixel-to-pixel non-uniformities. The sensor was tested for the detection of pH change of *E. coli* culture solution. As mentioned in Table 4, the sensitivity of this dual-mode sensor can be 26.2 mV/pH. The same group [37] proposed another potentiometric ISFET sensor with a subthreshold pH-to-time-to-voltage conversion scheme which introduced a programmable amplification factor to improve the sensitivity to 123.8 mV/pH. As mentioned in Table 4, the sensor showed a 0.01 pH resolution and small screening time (4 h) for *E. coli* detection. In another effort, Malpartida-Cardenas et al. [81] reported an LoC platform using ISFETs coupled with LAMP for fully-electronic detection of *P. falciparum*.

In an effort, Nikkhoo et al. [33] employed the ISFETs fabricated in conventional CMOS technology with additional polyvinylchloride (PVC)-based potassium-sensitive membrane and the specificity of bacteriophages as biological REs to implement an integrated platform with self-calibration capability for the detection of live bacteria in less than 10 min. Four strains of *E. coli* with two bacteriophages were used in the experiments at two different temperatures to show the sensor specificity and detection capability. Figure 11b shows the buffered readout circuit of this sensor for reading the source voltage (V_S_) of the ISFET. In another study, the same group [85] developed an all-electronic CMOS biosensor by using bacterial protein toxins, bacteriocins, as the biological REs for the detection of Gram-positive *S. aureus* and Gram-negative *E. coli* and in a short time (see Table 4).

Singh et al. [84] reported an aptamer-based field-effect transistor (aptaFET) device containing a gold interdigitated microelectrode (IDE) connected with an extended gate of FET. The DNA-aptamer is immobilized via gold-thiol chemistry on the IDE, as the RE against the malaria biomarker *P. falciparum* glutamate dehydrogenase in a serum sample. This biosensor demonstrated a short response time (~5 s), picomolar level-LoD (48.6 pM) in a broad dynamic range from 100 fM to 10 nM in diluted serum samples (Table 4).

### 3.2. Impedimetric, Capacitive and Conductometric Techniques

**Figure 12:**
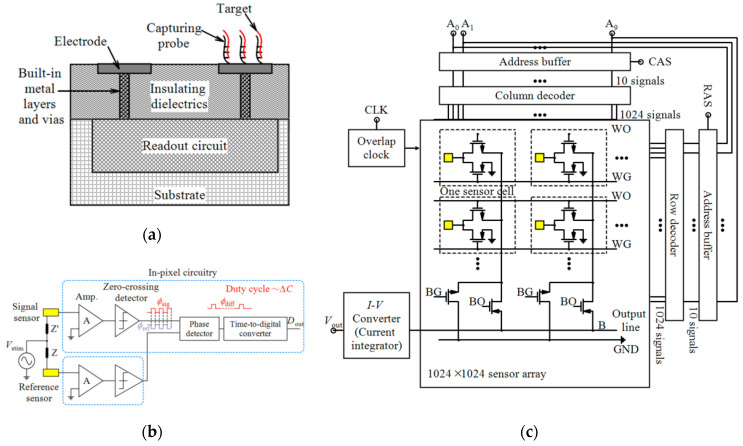
(**a**) General schematic of CMOS-based electrochemical sensors, (**b**) Architecture of in-pixel measurement in an electrochemical system by detecting the phase change between signal and reference electrodes, (**c**) Block diagram of an amperometric sensor.

Impedimetric, conductometric and capacitive biosensors process the frequency-dependent parameters of analyte like impedance, conductance and capacitance, respectively [96]. Impedimetric technique offers real-time label-free detection by measuring the resistance, capacitance and inductance information in the sample at different frequencies. This technique is a powerful approach for the detection of some biospecies like virus DNA [79] and bacterial cell [97] by monitoring the impedance transformations that occur as a result of selective binding of the biospecies to REs. These systems need media calibration, washing steps and noble metal electrodes [33]. The interfacing of electrochemical biosensors, as shown by a general schematic in Figure 12a, require sensing electrodes which can be implemented on the top metal layer of CMOS technology.

Lai et al. [79] used non-faradaic impedimetric for the detection of pathogenic avian influenza virus (AIV) oligonucleotides. In another effort, Couniot et al. [97] proposed a selectivity method by using lytic enzymes for impedimetric detection of whole-cell bacteria using atomic-layer-deposited (ALD)-Al_2_O_3_ passivated microelectrodes. This group [38] developed a CMOS oscillator-based capacitance to frequency converter integrated with Al/Al_2_O_3_ IDE which is suitable for whole bacterial cell detection in saline (high-conductive) buffers up to 575 MHz. Experiments by using *Staphylococcus epidermidis* (*S. epidermidis*) in physiological buffer verified the performance of this biosensor and after 20 min of bacterial incubation, it showed an LoD of 10^7^ CFU/mL. The same group [80] reported a 16 × 16 capacitive biosensor whose readout interface which converted capacitance to voltage based on charge sharing. Real-time detection of *S. epidermidis* binding events using this sensor demonstrated sensitivity of 2.18 mV/bacterial cell and an LoD of 7 bacteria/pixel. A 16 × 20 array electrochemical biosensor was reported by Hsu et al. [87] in which a polar-mode measurement method, as depicted in Figure 12b, and in-pixel digitization and accumulation (averaging) was employed.

Since the growth of bacteria forms capacitance variations in the proximity of the sensing electrodes, it can be monitored using a capacitive sensor. Ghafar-Zadeh et al. [26] proposed a core-CBCM (charge-based capacitance measurement) capacitive readout architecture technique to monitor the growth of bacteria in a medium. This sensor consists of a reference and a sensing IDE which are exposed to pure Luria–Bertani (LB) medium and *E. coli* suspended in the LB medium, respectively. They implemented the microfluidic channels using a direct-write fabrication process (DWFP) technique atop the electrodes. The experiments showed an LoD of 107 CFU/mL and a sensitivity of about 255 mV/fF (see Table 4). The same group [98] used bacteriophage as REs to detect *Salmonella* and *E. coli* and achieved a sensitivity of 29.3 mV/fF.

In another effort, Yao et al. [88] used a conductometric approach for bacteriophage-based bacteria detection. In comparison to PCR and ELISA, the use of phages allows for a rapid and simple sensing scheme. Since no biochemical reaction is involved in bacteriophage-based bacteria detection and no electrical charge is created, the amperometric method (explained in the next sub-section) is not suitable. On the other hand, the impedimetric method is complex. Conductometric method can be an alternative that only measures the resistance of the analyte between reference and working electrodes (RE and WE) and can be performed with DC stimulation. Yao et al. used T4 bacteriophages as REs to detect *E. coli*. Their sensor converts the sample resistance to frequency and a one-shot circuit is used to control the pulse-width of the output signal. This system is capable of monitoring bacteria with concentration ranging from 4 × 10^2^ to 4 × 10^4^ CFU/mL (see Table 4).

### 3.3. Other Electrochemical Techniques

Amperometric method measures the redox current generated during the reactions between the sample and the functionalized working electrode at a constant DC voltage. Similarly, a voltammetric sensor measures the current while the applied voltage is varied. The configuration of amperometric sensors is usually simpler to implement than impedimetric ones. However, this technique requires multiple steps to immobilize biological REs and provide redox currents as well as noble metals (like gold and platinum). Additionally, its specificity is affected by the interference from unknown chemicals [33]. 

Niitsu et al. [89] reported two types of electroless plated microelectrode arrays (MEAs) for direct bacteria and HeLa cell counting. A smaller size of these MEAs can make it possible to detect viruses (20–970 nm). An amperometry circuit is also reported. The cyclic voltammetry (CV) measurements by using the 4 × 4 array of 1.2 × 2.05 µm^2^-sized MEAs and the 16 × 16 array of 6 × 6 µm^2^-sized MEAs demonstrated the capability of these electroless-plated MEAs for the counting of bacterial-sized microbeads. For noise reduction, the same group [99] implemented a current integrator in conjunction with these bacterial-sized MEAs (see Figure 12c). They demonstrated the performance of the 4 × 4 array of their amperometric CMOS-based sensor and achieved an SNR of 30.4 dB. Their sensor was suitable for the detection of a single bacterial cell and counting their number in the range of a single cell to approximately 10^6^ cells. They developed their proposed sensor to a large-scale (1024 × 1024) array by employing two- 2P3M 0.6 µm CMOS technology [24].

Nanowire (NW) biosensors have attracted the attention of some researchers due to their high sensitivity and fast responses [100]. For instance, Huang et al. [90] used poly-Si NW to implement a CMOS wireless biosensor system-on-chip (bio-SSoC) for biomolecular detection of both cTnI and HBV DNA. DNA hybridization on NW leads the electrons in n-type doped poly-Si NW to be repelled from the surface and consequently, the resistance of the NW reduces which can be used for quantification of the DNA in the sample. 

Sun et al. [91] used a little-used technique called coulostatic discharge sensing for the detection of anti-Rubella and antiMumps antibodies in human serum and reported a 4096-pixel electrochemical biosensor array with IDEs surrounded by nanowells. The captured biospecies were transduced to a current and the sensor’s innate double-layer capacitance was discharged and then the measurements were converted to a voltage signal by each pixel. The three-dimensional (3D) trenches formed by opening the passivation across the entire IDE between two electrodes amplified the signal and increased collection efficiency.

## 4. Other Techniques

There are also some other CMOS-based biosensors like mechanical (such as surface acoustic wave (SAW) transducer [101] and cantilever [40,102]) and NMR-based biosensors [103], some of which are reported for the analysis of infectious diseases (as mentioned in Table 5).

### 4.1. Magnetic Sensors

**Figure 13:**
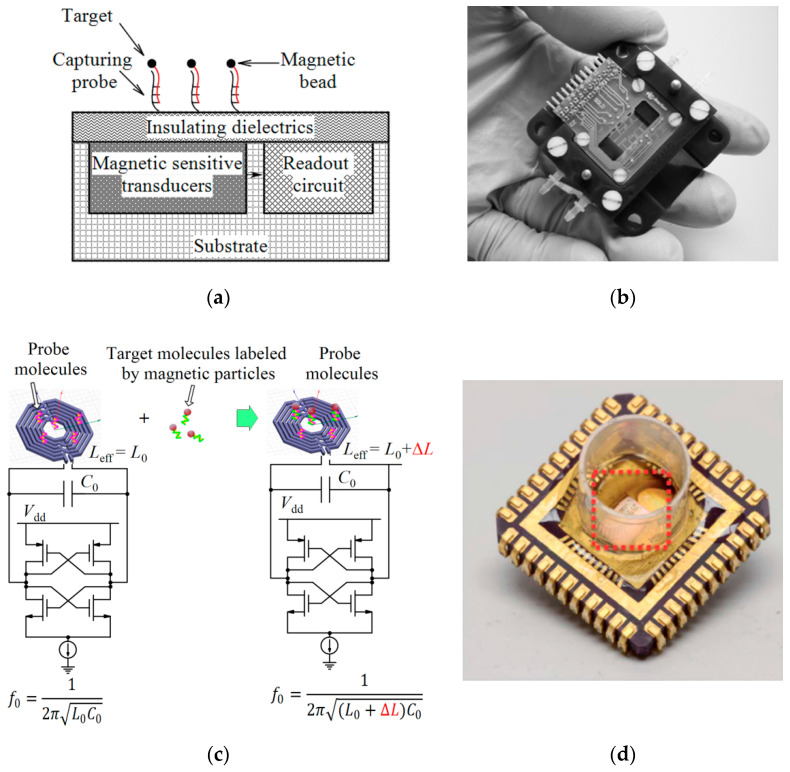
(**a**) Magnetic-based detection, (**b**) Packaged magnetoresistive immunosensor biosensor reported in [39], (**c**) Frequency-shift magnetic sensor sensing mechanism, (**d**) The disposable cartridge including an electrically connected magnetic-based biosensor chip inside a polypropylene well [105].

Magnetic biosensors detect the magnetism generated by the targets labeled with magnetic particles (see Figure 13a). Since the magnetic field can penetrate the insulating layers, transducers and samples are not in direct contact. So, the hardware preparation of the chip would be simplified before the assay [67]. Magnetic-based platforms provide the advantages of high sensitivity and in contrast to electrochemical biosensors, there is no virtual background because most biological samples do not have strong magnetic properties. Additionally, the same magnetic labels can also be employed for magnetic manipulation during sample preparation in the integrated system [105]. Ease of manufacturing and a wide range of magnetic bead diameters (from 50 nm to 20 µm) pave the way for different assays [104]. While the smaller ones are useful for research immunology for cell separation, larger beads conjugated with antibodies can be used for the improvement of the performance of ELISA by the separation of target analytes. 

To convert the sensed magnetism to an electrical signal, different transducers have been reported. Hall sensor as a fully CMOS-compatible device can be made of n-type silicon and convert the magnetic field to current or voltage signals. Based on Hall effect, the current carriers in a semiconductor can be deflected with the current flowing orthogonal to a magnetic field which applies a Lorentz force to the current carriers. So, charge deflection allows electronic detection [67]. A CMOS-based magnetic bead bioassay platform is reported by Aytur et al. [104] which is used for infectious disease diagnosis by immunological recognition similar to ELISA. The sensor chip contained a 1024-element array of Hall sensors which were bonded to a 25 × 15 mm^2^-PCB (see Table 5). A gold-plated 150-μL well on the PCB with a small hole at the bottom makes it possible to expose the sensor surface array to sample fluid. The biosensor is placed into the gap of a custom electromagnet core which can be operated in either an AC measurement mode or a DC washing mode. In the AC measurement mode, an excitation signal is generated and amplified by the laptop sound-card and a power amplifier, respectively, which induces the magnetic beads to produce local magnetic fields detectable by the sensor. In the DC washing mode, the electromagnet is powered by an adjustable power supply which can be used for removing specifically bound beads from the sensor surface. The experiments conducted on clinical serum samples verified the capability of this platform to capture the antigen of purified mouse IgG and detect human anti-dengue virus IgG.

Mujika et al. [39] reported a magnetoresistive immunosensor integrated with microfluidics for the detection of *E. coli* O157:H7 in food and clinical samples (see Figure 13b). A giant magnetoresistive multilayer structure played the role of a sensing film. Silicon nitride was selected as the sensor surface coating for the immobilization of antibodies to react with target antigens. This sensor could sense the small magnetic field variations caused by the presence of superparamagnetic beads bound to the antigens. The microfluidic network was made of an SU-8 photoresist channel network and a stereolithographic-based technique was used for packaging.

One of the magnetic techniques reported for biosensing applications is CMOS frequency-shift based magnetic resonant sensing. These sensors include LC resonators. When a magnetic bead is close to the inductor of the resonator, the oscillation frequency is decreased which can be electronically measured by counting the number of cycles over a fixed time interval. Pai et al. [105] proposed a frequency-shift based magnetic sensor for the detection of antigen and nucleic acid (see Figure 13c,d). The sensitivity of this sensor was increased by a noise reduction technique, CDS. They also proposed a magnetic freezing technique that neutralized the effect of magnetic beads on the sensor by saturating the magnetization of the beads with a small permanent magnet. By this technique, there was no need for a baseline measurement before the experimental assay, the SNR was enhanced, all the measurements could be done after the biological assay and the measurement time was reduced. The entire functionality of the sensor was integrated onto a reader PCB and a disposable open-well cartridge combined all sensor sites in a single reaction well without the need for microfluidic structure and pumps. The experiments showed this sensor can detect a 31 bp DNA oligomer with the concentration from 100 pM to 10 nM as well as an interferon-γ protein (relevant for tuberculosis diagnostics) with the concentration between 1 pM and 30 pM (see Table 5).

### 4.2. Mechanical Sensors

**Figure 14:**
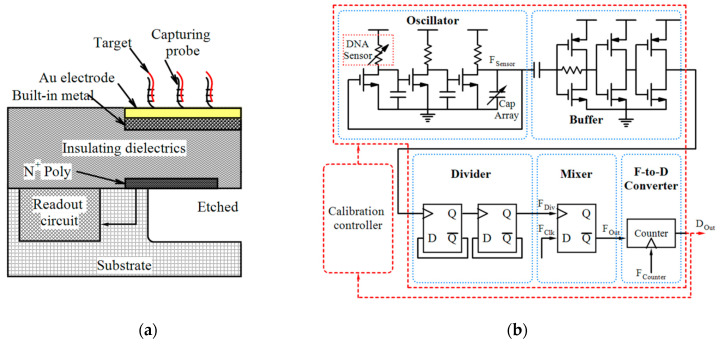
(**a**) Mechanical-based detection with cantilever; (**b**) Block diagram of a cantilever-based system on chip.

Surface-stress mechanical sensors are among the popular mechanical sensors used for biosensing. Binding of biomolecules to the functionalized surface of a miniature mechanical device, usually a cantilever, results in a measurable deflection of the device useful for sensing the concentration of the analyte (see Figure 14a). The cantilever might be designed for a static or a dynamic mode. In the former, only one surface of the cantilever is functionalized and can bend statically in one direction. In the latter, the entire cantilever is utilized to capture the targets and the attached mass affects its resonant frequency. So, based on the type of the sensor, resonant frequency [101], resistance [40], or optical [107] deflection of the cantilever [67] can be used for the detection of the attached mass.

Haung et al. [108] proposed a piezoresistive microcantilever-based biosensor for label-free and real-time determination of HBV DNA which transduced DNA hybridization into resistance changes. DNA hybridization process induces surface stress variations leading to the resistance changes of the embedded piezoresistor. After the standard construction of CMOS circuit (see Figure 14b), reactive ion etching (RIE) and biocompatibility of the post-process which was improved by gold deposition paved the way to acquire the identification tool. An oscillator-based self-calibration readout improved the functionality of the system. Experiments showed an LoD of 1 pM (Table 5).

## 5. Conclusions

Infectious diseases such as tuberculosis, hepatitis, HIV, Ebola, and COVID-19 constitute a major health threat worldwide. So, the research community needs a globally affordable method for the precise and timely diagnosis of these diseases. In this review, we have described various devices developed by using CMOS technology for the diagnosis and control of these diseases. CMOS technology offers great advantages such as high-throughput measurement, short time workflow, high resolution, low power consumption, parallel detection of a series of parameters, portability and automation. In recent years, various types of PoC testing, cellular and molecular analysis as well as remotely and mass screening measurements have been developed and adapted to CMOS-based devices to provide appropriate platforms useful for fighting against infectious diseases. Future studies are still required to achieve reliable and globally affordable systems to address this issue.

## Figures and Tables

**Table 1:** Some of the reported devices for COVID-19 applications.

Device	Target	Specifications	Ref.
Gr-FET based biosensor	SARS-CoV-2 spike protein and SARS-CoV-2	LoD in culture medium: 1.6 × 10^1^ pfu/mL LoD in clinical samples: 2.42 × 10^2^ copies/mL	[2]
Gr-FET immunosensor	COVID-19 spike protein S1	LoD: 0.2 pM Time to detect ~ 2 min	[3]
eCovSens-ultrasensitive PCB-based electrochemical device	nCovid-19 antigen, a spike protein domain 1 of SARS-CoV-2	By using screen printed carbon electrodes LoD: 90 fM Time to detect: 10–30 s	[8]
Dual-Functional Plasmonic Photothermal Biosensor	*RdRp*-COVID and *RdRp*-COVID-C	Combination of PPT and LSPR LoD: 0.22 pM	[9,10]
Cepheid (Xpert Xpress SARS-CoV-2 test)	*E* gene and *N* gene	Real-time RT-PCR sample-to-answer LoD: 250 copies/mL Time to result: 45 min	[15]
On-body skin-integrated sensor system	Key symptoms including cardiac- and respiratory-related signals, coughing and body temperature	A thin, soft sensor with a precision temperature sensor and a high-bandwidth accelerometer In direct mechanical communication with the skin overlying the suprasternal notch	[16,17]

**Table 2:** Advantages and disadvantages of CMOS technology.

Advantages	Disadvantages
- Very high levels of integration - Using both p-channel and n-channel devices - Low power - Good speed to power ratio - Fully restored logic levels - Rise and fall transition times of the same order - Very high noise-immunity and noise-margin - Extremely large fan-out capability - Excellent temperature stability and working over a wide temperature range - Low supply voltage ranges - Large logic swing	- Susceptible to damage by static electricity(it can be rectified by built-in protective devices or circuits) - Slower than bipolar technology - Leading-edge processes are not characterized for analog circuit design - In the scaling process, some second-order device characteristics, such as subthreshold operation, are usually paid less attention - Relatively high mismatch in CMOS devices

**Table 3:** CMOS-based optical biosensors reported for the diagnosis of various infectious diseases.

Optical Technique	Application	Tech.	Target	Area	Fluidic Structure	Array/Pixel #	LoD	Other Features	Ref.
Laser-induced fluorescence	Detection of *B. globigii* spores based on the combined use of ELISA and LIF detection	-	*B. globigii* spores	-	-	4 × 4	0.55 cells/probe	-	[52]
Spectrally-multiplexed FRET-on-a-Chip	Detection of marker gene sequences of *E. coli* and spinal muscular atropy disease	0.35 µm	Two DNA targets	(Optical filter area) 1.5 × 1.5 mm^2^	A PDMS ^1^ reservoir bonded to a glass cover slip	-	240 nM and 210 nM	AT ^2^ = 1 Sec/integration time	[53]
Fluorescence imaging	Mobile nucleic acid amplification testing for *C. trachomatis* screening using a smartphone CMOS sensor	-	*C. trachomatis* DNA	-	magnetofluidics	-	-	Sensitivity = 10^2^ to 10^3^ copies of molecular target	[46]
Fluorescence sensing in an integrated qPCR system	PoC diagnostics of *S. aureus*	0.35 µm CMOS	Target DNA	4 × 4 mm^2^	SU-8 microfluidics	7 × 8	-	Power = 33 mW (3.3 V)	[28]
Colorimetric-luminance readout for analysis of fluorescence signals	Simultaneous detection of Zika and chikungunya viral RNA via endpoint RT-LAMP and real-time LAMP detection of *N. gonorrhoeae* using a smartphone CMOS sensor	-	Zika and chikungunya *N. gonorrhoeae* DNA	-	-	1000	3.5 copies per 10 µL	-	[47]
Fluorescence imaging	Rapid and sensitive detection of *E. coli* O157:H7 using a smartphone CMOS sensor	-	*E. coli* O157:H7	-	Corning black flat-bottom 96-well plates	-	1 CFU/mL	AT = 2 h	[35]
Fluorescence imaging	Rapid telemonitoring system for Ebola and Marburg disease surveillance using a smartphone CMOS sensor	-	Ebola and Marburg	-	3M^TM^ polyester double-sided diagnostic microfluidic medical tape	-	-	AT = 20 min and 15 s Sensitivity ≈ pM ~ ng/mL	[48]
Fluorescence imaging	Simultaneous quantitative detection of *H. Pylori* using Qdots-labeled Immunochromatiographic test strips	-	*H. Pylori*	-	Test strip	-	5 mIU/mL	Specificity = 97% Sensitivity = 95%	[54]
Chemiluminescence/fluorescence imaging	Detection of *S. pneumoniae* ^3^ by the measurement of IgG antibody concentrations in human blood sera	0.5 µm	IgG antibody	-	-	4 × 8	-	-	[55]
Chemiluminescence imaging	Cross-flow immune chromatography based test for the detection of food-borne pathogens	-	*S. Typhimurium*	3 × 3 µm^2^	EOC cartridge	1.3 mega pixel	4.22 × 10^3^ CFU/mL	AT < 6 h	[25]
Chemiluminescence imaging	Immunoanalytical analysis for monitoring food contamination	-	*Vibrio* *parahaemolyticus*	3 × 3 µm^2^	EOC cartridge	1.3 mega pixel	1.4 × 10^4^ CFU/mL	-	[49]
Reader for LFIAs	Reader of LFIA for PoC diagnostics of Influenza A nucleoprotein	0.35 µm	Influenza A nucleoproteins	(Overall pixel array area) 12.28 mm^2^	-	4 × 64	0.5–200 ng/mL	FF ^4^ = 18% TORN ^5^ = 1.9 mV_rms_, Total = 21 µW Power/pixel = 0.32 µW	[56]
Shadow imaging	*E. coli* counting for sepsis diagnosis	-	*E. coli*	-	Microfluidics made of PMMA ^6^-DSA ^7^ layers and glass slides	-	-	-	[57]
Shadow imaging	ELISA measurements	-	*L. monocytogenes* and *S. typhimurium*	-	A commercial 96-well plate	5M pixel	Density of *L. monocytogenes* = 5 × 10^4^ cells/mL Density of *S. typhimurium* = 10^3^ cells/mL	-	[58]
Holographic on-chip microscope using fiber-optic arrays	Imaging human malaria parasites in thin blood smears	-	*P. falciparum*	-	Sample tray	-	-	Resolution < 1 µm FoV ≈ 24 mm^2^	[43]
Holographic on-chip microscope	Computational sensing of HSV	-	HSV	-	A reservoir	10M pixel	4 particles/mm^2^	FoV ≈ 30 mm^2^	[59]
Photon counting	PoC testing for the detection of HIV	-	HIV	-	-	376 × 314	10 fg/mL	-	[60]
Photon counting	Label-free quantitative immunoassay for Hepatitis B	-	HBV	-	-	376 × 314	10 fg/mL	-	[61]
Photon counting	LAMP technique for real-time DNA amplification and detection	-	*C. perfringens* (DNA)	5 × 5 mm^2^	Polypropylene cylindrical tube	376 × 314	1 fg/µL	AT < 1 h	[27]
SPR	Pathogen detection	-	*E. coli* and *S. aureus*	5.3 mm^2^	microfluidics made of PMMA-DSA-PMMA-DSA layers and a gold chip	500 × 582	10^5^ to 3.2 × 10^7^ CFUs/mL	-	[62]
SPR	Ultrasensitive detection of LAM with plasmonic grating biosensors in clinical samples of HIV negative patients with tuberculosis	-	Tuberculosis	-	ProPlate 24 well slide adapter	-	1 fg/mL	Sensitivity < 10 fM	[36]
Nano-plasmonic	One-step simultaneous detection of *C. trachomatis* and *N. gonorrhoeae* in urine	-	*C. trachomatis* and *N. gonorrhoeae*	-	PDMS microfluidics	-	LoD = 300 CFU/mL (for *C. trachomatis*) LoD = 1500 CFU/mL (for *N. gonorrhoeae*)	-	[63]
SP-IRIS	Enhanced light microscopy visualization of virus particles from Zika virus to filamentous ebolaviruses	-	Zika, Vesicular stomatitis, Vaccinia, Ebola	-	-	-	200,000 particles/mm^2^	-	[64]
Mach− Zehnder Interferometer	Detection of tuberculosis in urine samples using a nanophotonic PoC Platform	-	Tuberculosis	7.16 × 6.76 mm^2^	Polymer microfluidic cartridge	-	475 pg/mL (27.14 pM)	-	[65]
Optical microscopy using angular spatial frequency processing	An image cytometer based on angular spatial frequency processing for the detection of waterborne microorganisms	-	*E. Coli*, *L. pneumophila* and *Phytoplank*	5.70 × 4.28 mm^2^	A fluidic system comprising a barometric pump and a hollow fiber membrane filter	5M pixel	0.2 cells/mL	-	[42]

^1^ PDMS: poly-dimethylsiloxane, ^2^ AT: Analysis time, ^3^ *S. pneumoniae*: *Streptococcus pneumoniae*, ^4^ FF: Fill factor, ^5^ TORN: Total output referred noise, ^6^ PMMA: Polymethyl methacrylate, ^7^ DSA: Double-sided adhesive.

**Table 4:** CMOS-based electrochemical sensors reported for the diagnosis of various infectious diseases.

Technique	Application	Tech.	Target	Area	Fluidic Structure	Array/pixel #	Power	Other Features	Ref.
ISFET	*P. falciparum* malaria diagnosis and artemisinin-resistance detection	0.35 µm	*P. falciparum* DNA	0.56 mm^2^	Laser-cut acrylic microfluidic chamber	64 × 64	-	LoD (LAMP) = 1 Copies/reaction, LoD (pH-LAMP) = 10 Copies/reaction	[81]
ISFET	Simultaneous detection of independent DNA sequencing of three bacterial genomes	0.35 µm	Target DNA (of *Vibrio fischeri*, *E. Coli*, *R. palustris*)	17.5 × 17.5 mm^2^	polycarbonate flow cell	1.5 M, 7.2 M and 13 M	-	-	[82]
ISFET and optical	Genome diagnostics of *E. Coli*	0.18 μm	Target DNA	2.5 × 5 mm^2^	A 3D-printed plastic reservoir	64 × 64	105.6 mW	Sensitivity = 26.2 mV/pH FPNR ^1^ = 4% to 0.3% Speed = 1200 fps ^2^	[83]
ISFET	*E. coli* Screening	65 nm	*E. Coli*	5 × 5 mm^2^	PDMS cylindrical reservoir	512 × 128	Pixel array = 80.6 mW Analog blocks = 108.4 mW Digital blocks = 6.5 mW	Sensitivity = 123.8 mV/pH Resolution = 0.01 pH Density range = 14 to 140 CFU/mL Screening time = 4 h	[37]
AptaFET	Detection of *P. falciparum* glutamate dehydrogenase in serum samples	0.7 µm	*P. falciparum* glutamate dehydrogenase	-	-	-	-	LoD = 48.6 pM Dynamic range = 100 fM to 10 nM	[84]
Potassium-sensitive FET sensor	Detection of *E. Coli*	0.18 µm	*E. Coli*	1.5 × 0.6 mm^2^	A dark chamber	6	-	AT < 30 Min	[33]
Potassium-sensitive FET sensor	Rapid bacterial detection	0.13 µm	*S. aureus*, *E. Coli*	1.6 × 1.6 mm^2^	-	2	-	LoD = 10^3^ bacteria/mL	[85]
Impedimetric	AIV detection	0.35 µm 2P4M	Viral target DNA	-	-	4 × 4	-	LoD ~ 6.14 fg/mL	[86]
Impedimetric	Detection of Zika virus	0.18 µm	Zika Virus oligonucleotide	3 × 4 mm^2^	-	16 × 20	63 mW	-	[87]
Capacitive	Bacteria detection in saline buffers	0.25 µm	*S. epidermidis*	220 × 230 µm^2^	-	1	29 mW	LoD = 10 fF (10^7^ CFU/mL) Sensitivity = 11 kHz/fF (@ 254 MHz @ 17.5 pF)	[38]
Capacitive	Detection of single bacterial cell	0.25 µm	*S. epidermidis*	14 × 16 µm^2^	-	16 × 16	29 µW	LoD = 450 aF (~ 7 bact.) Sensitivity = 55 mV/fF (22 mV/bact.)	[80]
Capacitive	Bacteria growth monitoring	0.18 µm	*E. Coli*	100 × 100 µm^2^	DWFP microfluidics	1	-	LoD = 10^7^ CFU/mL Sensitivity = 255 mV/fF	[26]
Conductometric	Growth monitoring and sensing of bacteria	0.35 µm	*E. Coli*	190 × 220 µm^2^	ABP cultureware	8	1.85 mW	Concentration = 4 × 10^2^ to 4 × 10^4^ CFU/mL	[88]
Amperometric	Direct counting of bacterial and HeLa cells	0.6 µm	Bacteria-sized microbeads and HeLa cells	(for 1024 × 1024) 7.48 mm^2^	-	1024 × 1024, 4 × 4, 16 × 16	-	-	[89]
Amperometric	Bacteria Counting	0.6 µm 2P3M	Bacterial-sized microbeads	7.6 ×7.1 mm^2^	-	1024 × 1024	9.5 mW	Detection limit = 10^6^ cells	[24]
Poly-Si NW	Biomolecular detection	0.35 2p4M	HBV DNA and cTnI	2.5 × 2.5 mm^2^	A plastic reservoir	-	-	LoD = 10 fM (With post-etching)	[90]
Coulostatic discharge sensing	Rubella and mumps virus detection	0.18 µm	Rubella and mumps virus capsid protein	5 × 5 mm^2^	A well	64 × 64	95 mW	LoD = 100 nM	[91] ([92])

^1^ FPNR: Fixed pattern noise reduction, ^2^ fps: frames/second.

**Table 5:** Magnetic, mechanical and other CMOS-based devices reported for the diagnosis of various infectious diseases.

Technique	Application	Tech.	Target	Area	Fluidic Structure	Array/Pixel #	Power	Other Features	Ref.
Magnetic (Hall sensor)	Diagnosis of infectious disease (Dengue)	0.25 µm CMOS	Antigen of purified mouse IgG and human anti-dengue virus IgG	2.5 × 2.5 mm^2^	Integrated gold-plated 150-μL sample wells	1024	-	-	[104]
Magnetic (Frequency-shift based sensing)	Detection of an Interferon-γ protein (relevant for tuberculosis diagnostics) and a 31 bp DNA oligomer	0.13 µm CMOS	Interferon-γ protein and a 31 bp DNA oligomer	2.95 × 2.56 µm^2^	A polypropylene well	8	165 mW	LoD (Interferon-γ) = 1 pM, LoD (DNA) = 100 pM	[105] ([106])
Magnetic (Magnetoresistive)	Detection of the pathogen *E. coli* O157:H7 in food and clinical samples	-	*E. coli* O157:H7	16 × 21 mm^2^	4 parallel SU-8 microchannels	16 magnetoresistive meanders in groups of 4	-	Specificity = 10^5^ CFU/mL	[39]
Peizo-resistive (cantilever-based sensor)	HBV Detection	0.35 µm Bio-MEMS CMOS	19 base HBV DNA	30.4 mm^2^	-	18	Receive: 12.9 mW Transmit: 18.6 mW Sleep mode: 225 µW	LoD < 1 pM	[40]

## References

[1] Aytur T.S. (2007). A CMOS Biosensor for Infectious Disease Detection.

[2] Seo G., Lee G., Kim M.J., Baek S.-H., Choi M., Ku K.B., Lee C.-S., Jun S., Park D., Kim H.G. (2020). Rapid detection of COVID-19 causative virus (SARS-CoV-2) in human nasopharyngeal swab specimens using field-effect transistor-based biosensor. ACS Nano.

[3] Zhang X., Qi Q., Jing Q., Ao S., Zhang Z., Ding M., Wu M., Liu K., Wang W., Ling Y. (2020). Electrical probing of COVID-19 spike protein receptor binding domain via a graphene field-effect transistor. arXiv.

[4] Cardean Transistors™ Made Available to Companies and Government Agencies Willing to Build Handheld Coronavirus Detection Devices. https://www.technologynetworks.com/diagnostics/product-news/cardean-transistors-made-available-to-companies-and-government-agencies-willing-to-build-handheld-332759.

[5] Diagnostics with Molecular Scissors – Is This also Possible for on-site COVID-19 Tests?. https://www.gesundheitsindustrie-bw.de/en/article/news/Diagnostics-with-molecular-scissors-is-this-also-possible-for-on-site-COVID-19-tests.

[6] Hajian R., Balderston S., Tran T., DeBoer T., Etienne J., Sandhu M., Wauford N.A., Chung J.-Y., Nokes J., Athaiya M. (2019). Detection of unamplified target genes via CRISPR–Cas9 immobilized on a graphene field-effect transistor. Nat. Biomed. Eng..

[7] Bruch R., Baaske J., Chatelle C., Meirich M., Madlener S., Weber W., Dincer C., Urban G.A. (2019). CRISPR/Cas13a-powered electrochemical microfluidic biosensor for nucleic acid amplification-free miRNA diagnostics. Adv. Mater..

[8] Mahari S., Roberts A., Shahdeo D., Gandhi S. (2020). eCovSens-Ultrasensitive Novel In-House Built Printed Circuit Board Based Electrochemical Device for Rapid Detection of nCovid-19 antigen, a spike protein domain 1 of SARS-CoV-2. bioRxiv.

[9] Qiu G., Gai Z., Tao Y., Schmitt J., Kullak-Ublick G.A., Wang J. (2020). Dual-functional plasmonic photothermal biosensors for highly accurate severe acute respiratory syndrome coronavirus 2 detection. ACS Nano.

[10] A Biosensor for the COVID-19 Virus. https://tectales.com/wearables-sensors/a-biosensor-for-the-covid-19-virus.html.

[11] The HEMEMICS Platform Empowers Healthcare Providers to Rapidly Identify Infectious Disease Pathogens in 60 Seconds or Less and Generate Real-time Alerts to an Outbreak. https://hememics.com/.

[12] HEMEMICS Biotechnologies, Inc. Receives HHS Support to Develop Rapid Antigen, Antibody Diagnostic to Identify COVID-19 Infected Americans. https://www.newswise.com/coronavirus/hememics-biotechnologies-inc-receives-hhs-support-to-develop-rapid-antigen-antibody-diagnostic-to-identify-covid-19-infected-americans/?article_id=730032.

[13] Roswell Biotechnologies and Imec to Develop First Molecular Electronics Biosensor Chips for Infectious Disease Surveillance, Precision Medicine and DNA Storage. https://www.roswellbiotech.com/wp-content/uploads/2020/05/roswell-biotechnologies-and-imec-to-develop-first-molecular-electronics-biosensor-chips-for-infectious-disease.pdf.

[14] Moran A., Beavis K.G., Matushek S.M., Ciaglia C., Francois N., Tesic V., Love N. (2020). The detection of SARS-CoV-2 using the cepheid xpert xpress SARS-CoV-2 and Roche cobas SARS-CoV-2 assays. J. Clin. Microbiol..

[15] 4 Fast-track Covid-19 Diagnostic Tests. https://www.eetimes.eu/4-fast-track-covid-19-diagnostic-tests/.

[16] Jeong H., Rogers J.A., Xu S. (2020). Continuous on-body sensing for the COVID-19 pandemic: Gaps and opportunities. Sci. Adv..

[17] Monitoring COVID-19 from Hospital to Home: First Wearable Device Continuously Tracks Key Symptoms. https://news.northwestern.edu/stories/2020/04/monitoring-covid-19-from-hospital-to-home-first-wearable-device-continuously-tracks-key-symptoms/.

[18] Wong C.K., Ho D.T.Y., Tam A.R., Zhou M., Lau Y.M., Tang M.O.Y., Tong R.C.F., Rajput K.S., Chen G., Chan S.C. (2020). Artificial intelligence mobile health platform for early detection of COVID-19 in quarantine subjects using a wearable biosensor: protocol for a randomised controlled trial. BMJ Open.

[19] Zhao H., Liu F., Xie W., Zhou T.-C., OuYang J., Jin L., Li H., Zhao C.-Y., Zhang L., Wei J. (2020). Ultrasensitive supersandwich-type electrochemical sensor for SARS-CoV-2 from the infected COVID-19 patients using a smartphone. Sens. Actuators B Chem..

[20] Mao K., Zhang H., Yang Z. (2020). The potential of an integrated biosensor system with mobile health and wastewater-based epidemiology (iBMW) for the prevention, surveillance, monitoring and intervention of the COVID-19 pandemic. Biosens. Bioelectron..

[21] Chandra P. (2020). Miniaturized label-free smartphone assisted electrochemical sensing approach for personalized COVID-19 diagnosis. Sens. Int..

[22] Alvarez A. (1990). Introduction to BiCMOS. BiCMOS Technology and Applications.

[23] Nishi Y. (1981). Comparison of new technologies for VLSI: Possibilities and limitations. Microelectron. J..

[24] Gamo K., Nakazato K., Niitsu K. (2017). A current-integration-based CMOS amperometric sensor with 1024× 1024 bacteria-sized microelectrode array for high-sensitivity bacteria counting. IEICE Trans. Electron..

[25] Jeon J.-W., Kim J.-H., Lee J.-M., Lee W.-H., Lee D.-Y., Paek S.-H. (2014). Rapid immuno-analytical system physically integrated with lens-free CMOS image sensor for food-borne pathogens. Biosens. Bioelectron..

[26] Ghafar-Zadeh E., Sawan M., Chodavarapu V.P., Hosseini-Nia T. (2010). Bacteria growth monitoring through a differential CMOS capacitive sensor. IEEE Trans. Biomed. Circuits Syst..

[27] Wang T., Devadhasan J.P., Lee D.Y., Kim S. (2016). Real-time DNA amplification and detection system based on a CMOS image sensor. Anal. Sci..

[28] Norian H., Field R.M., Kymissis I., Shepard K.L. (2014). An integrated CMOS quantitative-polymerase-chain-reaction lab-on-chip for point-of-care diagnostics. Lab Chip.

[29] Van Dorst B., Brivio M., Van Der Sar E., Blom M., Reuvekamp S., Tanzi S., Groenhuis R., Adojutelegan A., Lous E.-J., Frederix F. (2016). Integration of an optical CMOS sensor with a microfluidic channel allows a sensitive readout for biological assays in point-of-care tests. Biosens. Bioelectron..

[30] Sun G., Nakayama Y., Dagdanpurev S., Abe S., Nishimura H., Kirimoto T., Matsui T. (2017). Remote sensing of multiple vital signs using a CMOS camera-equipped infrared thermography system and its clinical application in rapidly screening patients with suspected infectious diseases. Int. J. Infect. Dis..

[31] Zourob M., Elwary S., Turner A.P. (2008). Principles of Bacterial Detection: Biosensors, Recognition Receptors and Microsystems.

[32] Lazcka O., Del Campo F.J., Munoz F.X. (2007). Pathogen detection: A perspective of traditional methods and biosensors. Biosens. Bioelectron..

[33] Nikkhoo N., Gulak P.G., Maxwell K. (2013). Rapid detection of E. coli bacteria using potassium-sensitive FETs in CMOS. IEEE Trans. Biomed. Circuits Syst..

[34] Preechakasedkit P., Pinwattana K., Dungchai W., Siangproh W., Chaicumpa W., Tongtawe P., Chailapakul O. (2012). Development of a one-step immunochromatographic strip test using gold nanoparticles for the rapid detection of Salmonella typhi in human serum. Biosens. Bioelectron..

[35] Zeinhom M.M.A., Wang Y., Song Y., Zhu M.-J., Lin Y., Du D. (2018). A portable smart-phone device for rapid and sensitive detection of E. coli O157: H7 in Yoghurt and Egg. Biosens. Bioelectron..

[36] Wood A., Barizuddin S., Darr C.M., Mathai C.J., Ball A., Minch K., Somoskovi A., Hamasur B., Connelly J.T., Weigl B. (2019). Ultrasensitive detection of lipoarabinomannan with plasmonic grating biosensors in clinical samples of HIV negative patients with tuberculosis. PLoS ONE.

[37] Jiang Y., Liu X., Dang T.C., Huang X., Feng H., Zhang Q., Yu H. (2018). A High-Sensitivity Potentiometric 65-nm CMOS ISFET Sensor for Rapid E. coli Screening. IEEE Trans. Biomed. Circuits Syst..

[38] Couniot N., Bol D., Poncelet O., Francis L.A., Flandre D. (2014). A capacitance-to-frequency converter with on-chip passivated microelectrodes for bacteria detection in saline buffers up to 575 MHz. IEEE Trans. Circuits Syst. Ii: Express Briefs.

[39] Mujika M., Arana S., Castano E., Tijero M., Vilares R., Ruano-Lopez J., Cruz A., Sainz L., Berganza J. (2009). Magnetoresistive immunosensor for the detection of Escherichia coli O157: H7 including a microfluidic network. Biosens. Bioelectron..

[40] Huang Y.-J., Huang C.-W., Lin T.-H., Lin C.-T., Chen L.-G., Hsiao P.-Y., Wu B.-R., Hsueh H.-T., Kuo B.-J., Tsai H.-H. (2013). A CMOS cantilever-based label-free DNA SoC with improved sensitivity for hepatitis B virus detection. IEEE Trans. Biomed. Circuits Syst..

[41] Wang T., Kim S., An J.H. (2017). A novel CMOS image sensor system for quantitative loop-mediated isothermal amplification assays to detect food-borne pathogens. J. Microbiol. Methods.

[42] Pérez J.M., Jofre M., Martínez P., Yáñez M., Catalan V., Pruneri V. (2015). An image cytometer based on angular spatial frequency processing and its validation for rapid detection and quantification of waterborne microorganisms. Analyst.

[43] Bishara W., Sikora U., Mudanyali O., Su T.-W., Yaglidere O., Luckhart S., Ozcan A. (2011). Holographic pixel super-resolution in portable lensless on-chip microscopy using a fiber-optic array. Lab Chip.

[44] Moon S., Keles H.O., Ozcan A., Khademhosseini A., Hæggstrom E., Kuritzkes D., Demirci U. (2009). Integrating microfluidics and lensless imaging for point-of-care testing. Biosens. Bioelectron..

[45] Viswanath B., Kristine Y.M., Kim S. (2018). Recent trends in the development of complementary metal oxide semiconductor image sensors to detect foodborne bacterial pathogens. Trac Trends Anal. Chem..

[46] Shin D., Athamanolap P., Chen L., Hardick J., Lewis M., Hsieh Y.-H., Rothman R., Gaydos C.A., Wang T. (2017). Mobile nucleic acid amplification testing (mobiNAAT) for Chlamydia trachomatis screening in hospital emergency department settings. Sci. Rep..

[47] Priye A., Ball C.S., Meagher R.J. (2018). Colorimetric-luminance readout for quantitative analysis of fluorescence signals with a smartphone CMOS sensor. Anal. Chem..

[48] Natesan M., Wu S.-W., Chen C.-I., Jensen S.M., Karlovac N., Dyas B.K., Mudanyali O., Ulrich R.G. (2018). A smartphone-based rapid telemonitoring system for Ebola and Marburg disease surveillance. ACS Sens..

[49] Seo S.-M., Kim S.-W., Jeon J.-W., Kim J.-H., Kim H.-S., Cho J.-H., Lee W.-H., Paek S.-H. (2016). Food contamination monitoring via internet of things, exemplified by using pocket-sized immunosensor as terminal unit. Sens. Actuators B: Chem..

[50] Tsouti V., Boutopoulos C., Zergioti I., Chatzandroulis S. (2011). Capacitive microsystems for biological sensing. Biosens. Bioelectron..

[51] Göröcs Z., Ozcan A. (2012). On-chip biomedical imaging. IEEE Rev. Biomed. Eng..

[52] Song J.M., Culha M., Kasili P.M., Griffin G.D., Vo-Dinh T. (2005). A compact CMOS biochip immunosensor towards the detection of a single bacteria. Biosens. Bioelectron..

[53] Ho D., Noor M.O., Krull U.J., Gulak G., Genov R. (2013). CMOS spectrally-multiplexed Fret-on-a-Chip for DNA analysis. IEEE Trans. Biomed. Circuits Syst..

[54] Zheng Y., Wang K., Zhang J., Qin W., Yan X., Shen G., Gao G., Pan F., Cui D. (2016). Simultaneous quantitative detection of Helicobacter pylori based on a rapid and sensitive testing platform using quantum dots-labeled immunochromatiographic test strips. Nanoscale Res. Lett..

[55] Baader J., Klapproth H., Bednar S., Brandstetter T., Rühe J., Lehmann M., Freund I. (2011). Polysaccharide microarrays with a CMOS based signal detection unit. Biosens. Bioelectron..

[56] Pilavaki E., Valente V., Demosthenous A. (2018). CMOS Image Sensor for Lateral Flow Immunoassay Readers. IEEE Trans. Circuits Syst. Ii Express Briefs.

[57] Moon S., Manzur F., Manzur T., Klapperich C., Demirci U. (2009). Escherichia coli counting using lens-free imaging for sepsis diagnosis. Proceedings of Unmanned/Unattended Sensors and Sensor Networks.

[58] Lee J., Kwak Y.H., Paek S.-H., Han S., Seo S. (2014). CMOS image sensor-based ELISA detector using lens-free shadow imaging platform. Sens. Actuators B: Chem..

[59] Ray A., Daloglu M.U., Ho J., Torres A., Mcleod E., Ozcan A. (2017). Computational sensing of herpes simplex virus using a cost-effective on-chip microscope. Sci. Rep..

[60] Devadhasan J.P., Kim S. (2013). CMOS image sensor based HIV diagnosis: a smart system for point-of-care approach. Biochip J..

[61] Devadhasan J.P., Kim S. (2015). Label free quantitative immunoassay for Hepatitis B. J. Nanosci. Nanotechnol..

[62] Tokel O., Yildiz U.H., Inci F., Durmus N.G., Ekiz O.O., Turker B., Cetin C., Rao S., Sridhar K., Natarajan N. (2015). Portable microfluidic integrated plasmonic platform for pathogen detection. Sci. Rep..

[63] Soler M., Belushkin A., Cavallini A., Kebbi-Beghdadi C., Greub G., Altug H. (2017). Multiplexed nanoplasmonic biosensor for one-step simultaneous detection of Chlamydia trachomatis and Neisseria gonorrhoeae in urine. Biosens. Bioelectron..

[64] Daaboul G.G., Freedman D.S., Scherr S.M., Carter E., Rosca A., Bernstein D., Mire C.E., Agans K.N., Hoenen T., Geisbert T.W. (2017). Enhanced light microscopy visualization of virus particles from Zika virus to filamentous ebolaviruses. PLoS ONE.

[65] Ramirez-Priego P., Martens D., Elamin A.A., Soetaert P., Van Roy W., Vos R., Anton B., Bockstaele R., Becker H., Singh M. (2018). Label-free and real-time detection of tuberculosis in human urine samples using a nanophotonic point-of-care platform. ACS Sens..

[66] Velasco-Garcia M. (2009). Optical biosensors for probing at the cellular level: A review of recent progress and future prospects. Proceedings of Seminars in Cell & Developmental Biology.

[67] Lei K.-M., Mak P.-I., Law M.-K., Martins R.P. (2016). CMOS biosensors for in vitro diagnosis–transducing mechanisms and applications. Lab Chip.

[68] Jang B., Cao P., Chevalier A., Ellington A., Hassibi A. A CMOS fluorescent-based biosensor microarray. Proceedings of the 2009 IEEE International Solid-State Circuits Conference-Digest of Technical Papers.

[69] Ta-chien D.H., Sorgenfrei S., Gong P., Levicky R., Shepard K.L. (2009). A 0.18-µm CMOS Array Sensor for Integrated Time-Resolved Fluorescence Detection. IEEE J. Solid-State Circuits.

[70] Hong L., McManus S., Yang H., Sengupta K. A fully integrated CMOS fluorescence biosensor with on-chip nanophotonic filter. Proceedings of the 2015 Symposium on VLSI Circuits (VLSI Circuits).

[71] Erill I., Campoy S., Rus J., Fonseca L., Ivorra A., Navarro Z., Plaza J.A., Aguiló J., Barbé J. (2004). Development of a CMOS-compatible PCR chip: comparison of design and system strategies. J. Micromech. Microeng..

[72] Toumazou C., Shepherd L.M., Reed S.C., Chen G.I., Patel A., Garner D.M., Wang C.-J.A., Ou C.-P., Amin-Desai K., Athanasiou P. (2013). Simultaneous DNA amplification and detection using a pH-sensing semiconductor system. Nat. Methods.

[73] Sandeau L., Vuillaume C., Contié S., Grinenval E., Belloni F., Rigneault H., Owens R.M., Fournet M.B. (2015). Large area CMOS bio-pixel array for compact high sensitive multiplex biosensing. Lab Chip.

[74] Arandian A., Bagheri Z., Ehtesabi H., Najafi Nobar S., Aminoroaya N., Samimi A., Latifi H. (2019). Optical Imaging Approaches to Monitor Static and Dynamic Cell-on-Chip Platforms: A Tutorial Review. Small.

[75] Fan X., White I.M., Shopova S.I., Zhu H., Suter J.D., Sun Y. (2008). Sensitive optical biosensors for unlabeled targets: A review. Anal. Chim. Acta.

[76] Damborský P., Švitel J., Katrlík J. (2016). Optical biosensors. Essays Biochem..

[77] Garcia-Sucerquia J., Xu W., Jericho S.K., Klages P., Jericho M.H., Kreuzer H.J. (2006). Digital in-line holographic microscopy. Appl. Opt..

[78] Matsui T., Hakozaki Y., Suzuki S., Usui T., Kato T., Hasegawa K., Sugiyama Y., Sugamata M., Abe S. (2010). A novel screening method for influenza patients using a newly developed non-contact screening system. J. Infect..

[79] Lai W.-A., Lin C.-H., Yang Y.-S., Lu M.S.-C. (2012). Ultrasensitive and label-free detection of pathogenic avian influenza DNA by using CMOS impedimetric sensors. Biosens. Bioelectron..

[80] Couniot N., Francis L.A., Flandre D. (2016). A 16×16 CMOS Capacitive Biosensor Array Towards Detection of Single Bacterial Cell. IEEE Trans. Biomed. Circuits Syst..

[81] Malpartida-Cardenas K., Miscourides N., Rodriguez-Manzano J., Yu L.-S., Moser N., Baum J., Georgiou P. (2019). Quantitative and rapid Plasmodium falciparum malaria diagnosis and artemisinin-resistance detection using a CMOS Lab-on-Chip platform. Biosens. Bioelectron..

[82] Rothberg J.M., Hinz W., Rearick T.M., Schultz J., Mileski W., Davey M., Leamon J.H., Johnson K., Milgrew M.J., Edwards M. (2011). An integrated semiconductor device enabling non-optical genome sequencing. Nature.

[83] Huang X., Yu H., Liu X., Jiang Y., Yan M., Wu D. (2015). A dual-mode large-arrayed CMOS ISFET sensor for accurate and high-throughput pH sensing in biomedical diagnosis. IEEE Trans. Biomed. Eng..

[84] Singh N.K., Thungon P.D., Estrela P., Goswami P. (2019). Development of an aptamer-based field effect transistor biosensor for quantitative detection of Plasmodium falciparum glutamate dehydrogenase in serum samples. Biosens. Bioelectron..

[85] Nikkhoo N., Cumby N., Gulak P.G., Maxwell K.L. (2016). Rapid Bacterial Detection via an All-Electronic CMOS Biosensor. PLoS ONE.

[86] Lai W.-A., Lin C.-H., Yang Y.-S., Lu M.S.-C. Ultrasensitive detection of avian influenza virus by using CMOS impedimetric sensor arrays. Proceedings of the 2012 IEEE 25th International Conference on Micro Electro Mechanical Systems (MEMS).

[87] Hsu C.-L., Sun A., Zhao Y., Aronoff-Spencer E., Hall D.A. A 16 × 20 electrochemical CMOS biosensor array with in-pixel averaging using polar modulation. Proceedings of the 2018 IEEE Custom Integrated Circuits Conference (CICC).

[88] Yao L., Lamarche P., Tawil N., Khan R., Aliakbar A.M., Hassan M.H., Chodavarapu V.P., Mandeville R. (2011). CMOS conductometric system for growth monitoring and sensing of bacteria. IEEE Trans. Biomed. Circuits Syst..

[89] Niitsu K., Ota S., Gamo K., Kondo H., Hori M., Nakazato K. (2015). Development of microelectrode arrays using electroless plating for CMOS-based direct counting of bacterial and HeLa cells. IEEE Trans. Biomed. Circuits Syst..

[90] Huang C.-W., Huang Y.-J., Yen P.-W., Tsai H.-H., Liao H.-H., Juang Y.-Z., Lu S.-S., Lin C.-T. (2013). A CMOS wireless biomolecular sensing system-on-chip based on polysilicon nanowire technology. Lab Chip.

[91] Sun A.C., Alvarez-Fontecilla E., Venkatesh A., Aronoff-Spencer E., Hall D.A. (2018). High-density redox amplified coulostatic discharge-based biosensor array. IEEE J. Solid-State Circuits.

[92] Sun A., Alvarez-Fontecilla E., Venkatesh A., Aronoff-Spencer E., Hall D.A. A 64 × 64 high-density redox amplified coulostatic discharge-based biosensor array in 180 nm CMOS. Proceedings of the ESSCIRC 2017-43rd IEEE European Solid State Circuits Conference.

[93] Pourciel-Gouzy M., Sant W., Humenyuk I., Malaquin L., Dollat X., Temple-Boyer P. (2004). Development of pH-ISFET sensors for the detection of bacterial activity. Sens. Actuators B Chem..

[94] Bergveld P. (1970). Development of an ion-sensitive solid-state device for neurophysiological measurements. IEEE Trans. Biomed. Eng..

[95] Bausells J., Carrabina J., Errachid A., Merlos A. (1999). Ion-sensitive field-effect transistors fabricated in a commercial CMOS technology. Sens. Actuators B: Chem..

[96] Ghafar-Zadeh E. (2015). Wireless integrated biosensors for point-of-care diagnostic applications. Sensors.

[97] Couniot N., Vanzieleghem T., Rasson J., Van Overstraeten-Schlögel N., Poncelet O., Mahillon J., Francis L., Flandre D. (2015). Lytic enzymes as selectivity means for label-free, microfluidic and impedimetric detection of whole-cell bacteria using ALD-Al_2_O_3_ passivated microelectrodes. Biosens. Bioelectron..

[98] Yao L., Hajj-Hassan M., Ghafar-Zadeh E., Shabani A., Chodavarapu V., Zourob M. CMOS capactive sensor system for bacteria detection using phage organisms. Proceedings of the Canadian Conference on Electrical and Computer Engineering CCECE.

[99] Gamo K., Nakazato K., Niitsu K. (2016). Design, theoretical analysis, and experimental verification of a CMOS current integrator with 1.2 × 2.05 µm^2^ microelectrode array for high-sensitivity bacterial counting. Jpn. J. Appl. Phys..

[100] Ambhorkar P., Wang Z., Ko H., Lee S., Koo K.-i., Kim K., Cho D.-i.D. (2018). Nanowire-based biosensors: From growth to applications. Micromachines.

[101] Tigli O., Bivona L., Berg P., Zaghloul M.E. (2009). Fabrication and characterization of a surface-acoustic-wave biosensor in CMOS technology for cancer biomarker detection. IEEE Trans. Biomed. Circuits Syst..

[102] Li K.-W., Yen Y.-K. (2019). Gentamicin drug monitoring for peritonitis patients by using a CMOS-BioMEMS-based microcantilever sensor. Biosens. Bioelectron..

[103] Liu Y. (2008). CMOS Magnetic Cell Manipulator and CMOS NMR Biomolecular Sensor.

[104] Aytur T., Foley J., Anwar M., Boser B., Harris E., Beatty P.R. (2006). A novel magnetic bead bioassay platform using a microchip-based sensor for infectious disease diagnosis. J. Immunol. Methods.

[105] Pai A., Khachaturian A., Chapman S., Hu A., Wang H., Hajimiri A. (2014). A handheld magnetic sensing platform for antigen and nucleic acid detection. Analyst.

[106] Wang H., Chen Y., Hassibi A., Scherer A., Hajimiri A. A frequency-shift CMOS magnetic biosensor array with single-bead sensitivity and no external magnet. Proceedings of the Solid-State Circuits Conference-Digest of Technical Papers.

[107] Chen C.-H., Hwang R.-Z., Huang L.-S., Lin S.-M., Chen H.-C., Yang Y.-C., Lin Y.-T., Yu S.-A., Lin Y.-S., Wang Y.-H. (2008). A wireless bio-MEMS sensor for C-reactive protein detection based on nanomechanics. IEEE Trans. Biomed. Eng..

[108] Huang C.-W., Hsueh H.-T., Huang Y.-J., Liao H.-H., Tsai H.-H., Juang Y.-Z., Lin T.-H., Lu S.-S., Lin C.-T. (2013). A fully integrated wireless CMOS microcantilever lab chip for detection of DNA from Hepatitis B virus (HBV). Sens. Actuators B Chem..

